# Supplementary material for: Dissecting tumor microenvironment from spatially resolved transcriptomics data by heterogeneous graph learning
Source: Nat Commun. 2024 Jun 13;15:5057. doi: 10.1038/s41467-024-49171-7 (PMC11176411; doi:10.1038/s41467-024-49171-7)
Supplement: Supplementary file 1 — Supplementary information [file 41467_2024_49171_MOESM1_ESM.pdf]

Supplementary Materials of

Dissecting tumor ecosystems from spatially resolved transcriptomics data by heterogeneous graph learning

Chunman Zuo[1,2*], Junjie Xia[1,3], and Luonan Chen[4,5,6*]

[1] Institute of Artificial Intelligence, Shanghai Engineering Research Center of Industrial Big Data and Intelligent System, Donghua University, Shanghai 201620, China

[2] Key Laboratory of Symbolic Computation and Knowledge Engineering of Ministry of Education, Jilin University, Changchun 130022, China

[3] Department of Applied Mathematics, Donghua University, Shanghai 201620, China

[4] Key Laboratory of Systems Biology, Shanghai Institute of Biochemistry and Cell Biology, Center for Excellence in Molecular Cell Science, Chinese Academy of Sciences, Shanghai 200031, China

[5] Key Laboratory of Systems Health Science of Zhejiang Province, School of Life Science, Hangzhou Institute for Advanced Study, University of Chinese Academy of Sciences, Chinese Academy of Sciences, Hangzhou 310024, China

[6] West China Biomedical Big Data Center, Med-X center for informatics, West China Hospital, Sichuan University, Chengdu 610041, China

Supplementary Notes

Supplementary Note 1. Evaluation of cluster specificity and shareability for inferred cell-cell communications

We introduced a Gini index (GI)-based metric to assess the degree of inequality among different clusters in the distribution of CCC inferred by stKeep. Specifically, for each LRP, we computed the average interaction strength within each cluster, and then calculated GI for each LRP using 'gini' function from the reldist package [1]. This allowed us to evaluate the inequality level of CCC across different clusters. To provide a comprehensive comparison, we applied this statistical measure to analyze various entities including the corresponding used ligands and receptors, housekeeping genes, known layer-specific genes, and all expressed ligands and receptors, on 12 slices of the human DLPFC dataset. In summary, we found that (1) the GI score of CCC is marginally lower than the higher score for the relevant ligand and receptor within each LRP, and yet slightly surpasses the scores of the individual ligand and receptor. This trend agrees with the biological principle that CCC activation depends on the simultaneous expression of ligand and receptor for each LRP; (2) the GI distribution of the used ligands and receptors is the same as that of all expressed ligands and receptors; and (3) the GI distribution of CCC falls between that of housekeeping genes and that of layer-specific genes, suggesting that CCC exhibits both cluster-specificity and commonality. On average, approximately 31% of the LRPs display a GI score exceeding the mean value of layer-specific genes, while 15% exhibit a GI score below the mean of housekeeping genes. Overall, these results indicated that stKeep is able to identify cluster-specific and shared CCC patterns (Supplementary Fig.5a-e).

Supplementary Note 2. Dissecting heterogeneous cell populations on the TNBC sample by scRNA-seq data

To clearly clarify the heterogeneous tissue within cluster 9 on TNBC sample, we estimated the distribution of different cell types by mapping the paired scRNA-seq data using GraphST [2]. By such analysis, we found that (1) the lymphocyte region annotated in cluster 9 comprises not only T cells but also a large number of stromal cells. This mixed composition explains why stKeep identifies these regions as a cluster; and (2) other SRT methods are unable to identify distinct sub-regions within the lymphocyte region. To analyze heterogeneous cell populations within the stroma, adipose and lymphocyte regions, we conducted a detailed clustering analysis based on cell proportions of T and stromal cells, identifying three sub-clusters. To further

validate the accuracy, we conducted gene enrichment analysis of the up-regulated genes of each sub-cluster using DAVID (https://david.ncifcrf.gov/tools.jsp). Three sub-clusters show different functions: sub-cluster 1 cells are related to extracellular matrix organization and collagen degradation, potentially induced by infiltrating stromal cells; sub-cluster 2 cells participate in MAP kinase activation and overexpress *VEGFA* and *FGFR1*, potentially regulating endothelial cells response to stress; and sub-cluster 3 cells exhibit diverse functions such as T cell receptor signaling pathway, T cell activation, and Th1 and Th2 cell differentiation (Supplementary Fig.10a-e).

Supplementary Note 3. Individual graph component ablation analysis within cell module and gene module

We conducted extensive experiments to assess the impact of each graph on the performance of cell-modules and gene-modules using 12 slices from human DLPFC dataset. Specifically, (1) for each graph within the cell module, we evaluated its contribution to the identification of spatial clustering by removing the graph, and measured its impact using ASW. The lower the ASW, the higher the contribution of the graph. The comparison results revealed the following order of contribution, from most to least: spatial location graph (SLG), cell-region graph (CRG), histological similarity graph (HSG), transcriptomics similarity graph (TSG), and cell-gene graph (CGG). Notably, while the removal of CGG results in tighter clustering, it fails to accurately detect boundaries in finer structures (e.g., cluster 1 in slice 151669). In line with the findings from the DLPFC dataset, CGG demonstrates an important role in accurately detecting boundaries within cancer samples, notably highlighting cluster 33 in IDC and cluster 17 in BAS1, which is visually marked by black outlines. These results confirmed that our cell-module's structure is well designed and it enables to detect cell heterogeneity from different histological regions; and (2) for each graph within the gene module, we assessed its contribution to the learning of gene embeddings by removing the graph, and measured this influence by calculating the distance between the gene pairs identified from gene-modules using stKeep. The shorter the distance, the higher the contribution of the graph. The comparison results showed the order of contribution, from most to least: gene-cell state graph (GSG), gene-cell graph (GCG), PPI, and GRN, where PPI and GRN have a comparable performance (Supplementary Figs.13a-c and 14a).

Supplementary Note 4. Evaluation the robustness of stKeep in the presence of incomplete or incorrect prior gene-gene interactions

We evaluated the robustness of stKeep by computing the false positive rate (FPR) of the predicted gene-gene relations for 12 slices in the human DLPFC dataset. We assessed the impact of incomplete or incorrect prior gene-gene interactions (PPI and GRN) by randomly removing or adding gene-gene interactions (ranging from 10% to 90%) from the initial gene-gene interactions as inputs for the gene module. We considered the identified gene-gene relations from the initial complete set as the ground truth, and calculated FPR. Notably, stKeep demonstrates robustness, showcasing an FPR of zero for incomplete graphs and around 0.2% for incorrect prior interactions. Moreover, we introduced the recover edge rate (RER) to measure the proportion of the removed gene-gene interactions that can be recovered from gene-modules trained using incomplete prior graphs at different scales. On average, stKeep is able to recover 23% of the gene-gene relations. In addition, we calculated the Pearson correlation of LRP between spots within each cluster by CCC modules trained on varying proportions of incomplete LRPs. The results consistently demonstrated the ability of stKeep to produce similar outcomes across different proportions of prior LRPs. As a suggestion, users are advised to input confident LRP to infer their interaction strength (Supplementary Figs.14b-e).

Supplementary Note 5. Evaluation of the impact of histological regions on cell modules, gene modules, and CCC modules

To examine the impact of various histological regions on learning cell-modules, we utilized stKeep for analyzing two cancer samples (IDC and BAS1) trained with varying numbers of histological regions. Our findings revealed that the majority of clustering results exhibit high consistency, indicating the robustness of stKeep to different histological region inputs. Clearly, leveraging our defined 14 histological regions in both samples as input, stKeep demonstrates an enhanced ability to detect finer cancer cell-states, e.g., cluster 34 in IDC and cluster 20 in BAS1. In addition, we conducted an analysis without incorporating histological regions, using spatial nearest neighbors as positive samples while treating others as negative samples. This analysis result demonstrated that stKeep exhibits comparable performance when compared to models using histological regions as input. However, the inclusion of histological regions enhances the clarity of clustering boundaries, e.g., cluster 10 in IDC and cluster 16 in BAS1 (Supplementary Figs. 15a and 16a).

To further illustrate the robustness of stKeep in identifying cell-modules with varying histological region inputs and their impact on gene-modules and CCC modules, we conducted an analysis on four slices (151673-151676) of human DLPFC dataset. We used three different

types of regions as the input of stKeep: five regions (WM, Layer 6, Layer 5, Layer 4, and a region containing Layers 3, 2, and 1), six regions (WM, Layer 6, Layer 5, Layer 4, Layer 3, and a region containing Layers 2, and 1), and six regions (Layer 5, Layer 4, Layer 3, Layer 2, Layer 1, and a region containing WM and Layer 6). Our comprehensive comparison results revealed that (i) in the cell module, stKeep enables dissecting heterogeneous cell populations from the provided histological regions, which is consistent with the findings from the cancer samples; (ii) regarding gene-modules learned from predicted cell-states, they exhibit cell-state specific patterns, with most of them showing region-specific patterns based on gene expression levels between the input regions. More than 70% of gene-modules in the three types of heterogeneous region exhibit layer-specific patterns; (iii) in the CCC module, the Pearson correlation of CCC between spots within a cluster is comparable to that inferred from positive pairs based on annotated seven layers, and is significantly higher than that from randomly selected spot pairs. Additionally, over 75% of cluster-specific LRPs are consistent with the layer-specific LRPs (Supplementary Fig.17a-d).

In summary, we assert that the selection of positive and negative samples encourages cells/spots from the same region to be clustered closely in the latent space, thereby facilitating sub-clustering within these regions while maintaining overall cell heterogeneity. Furthermore, while coarse annotations of histological regions may sacrifice some intra-regional heterogeneity, they do not affect the identification of gene-modules and LRPs across different regions.

Supplementary Note 6. Training strategy for processing NSCLC sample

We adopted the following steps to analyze NSCLC sample. Specifically, we (1) trained the stKeep model by sequentially inputting SRT data from each FOV, and then utilized a batch size of 13K to subsample cells from 30 FOVs for training the model; and (2) iteratively utilized the strategy detailed in (1) to train the model until convergence was achieved. The entire training process of the NSCLC sample consumed 100 minutes and 6GB of memory.

Supplementary Figures

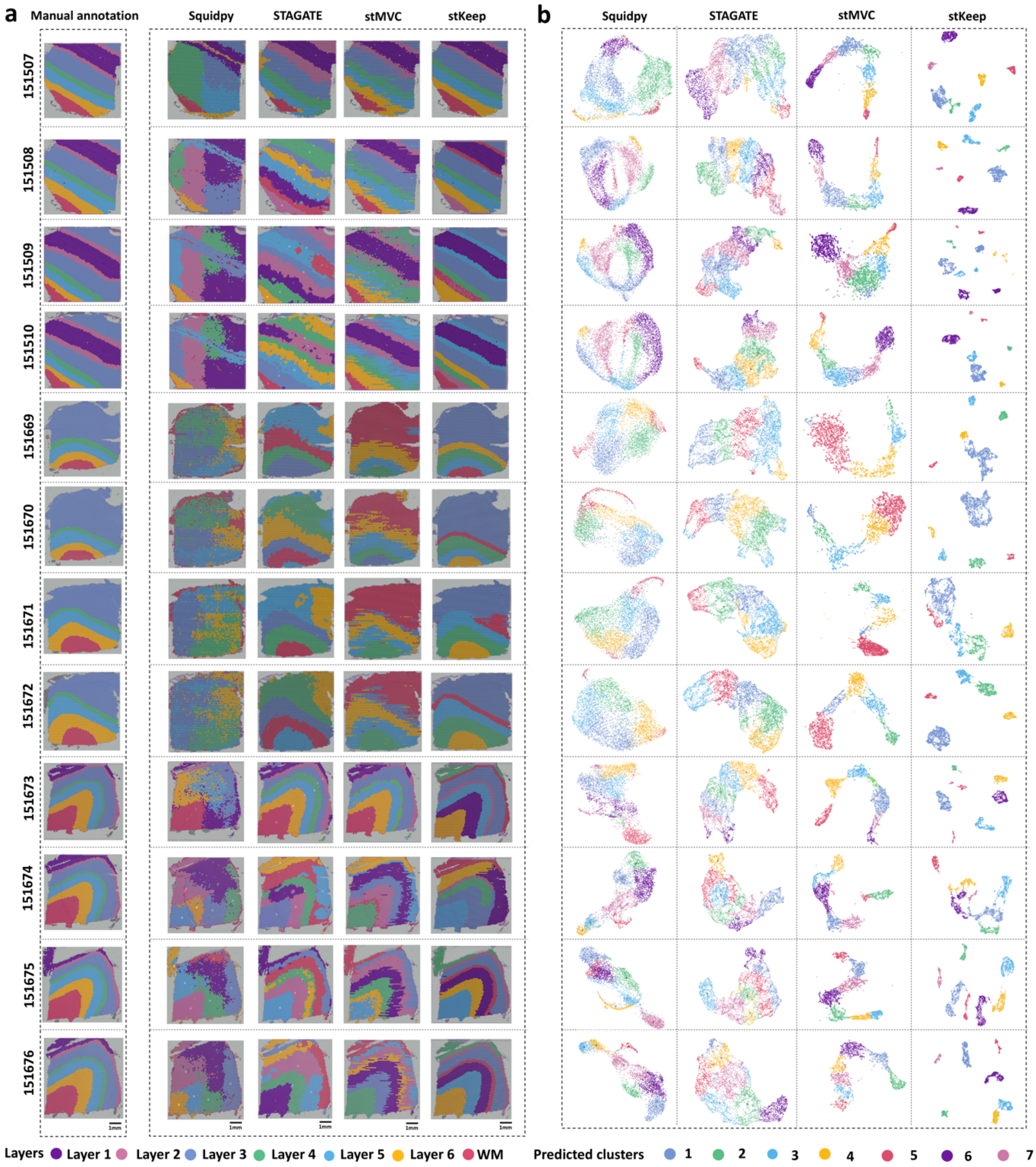

Supplementary Figure 1. Method comparison on 12 slices of the human DLPFC dataset. **a** Spatial clustering annotated by previous study [3], and detected by Squidpy, STAGATE, stMVC, and stKeep. Each cluster is indicated by a color. **b** UMAP visualization of the latent features by Squidpy, STAGATE, stMVC, and stKeep. For each method on each slice, the predicted cell clusters and their colors are the same as in **a**. Source data are provided as a Source Data file.

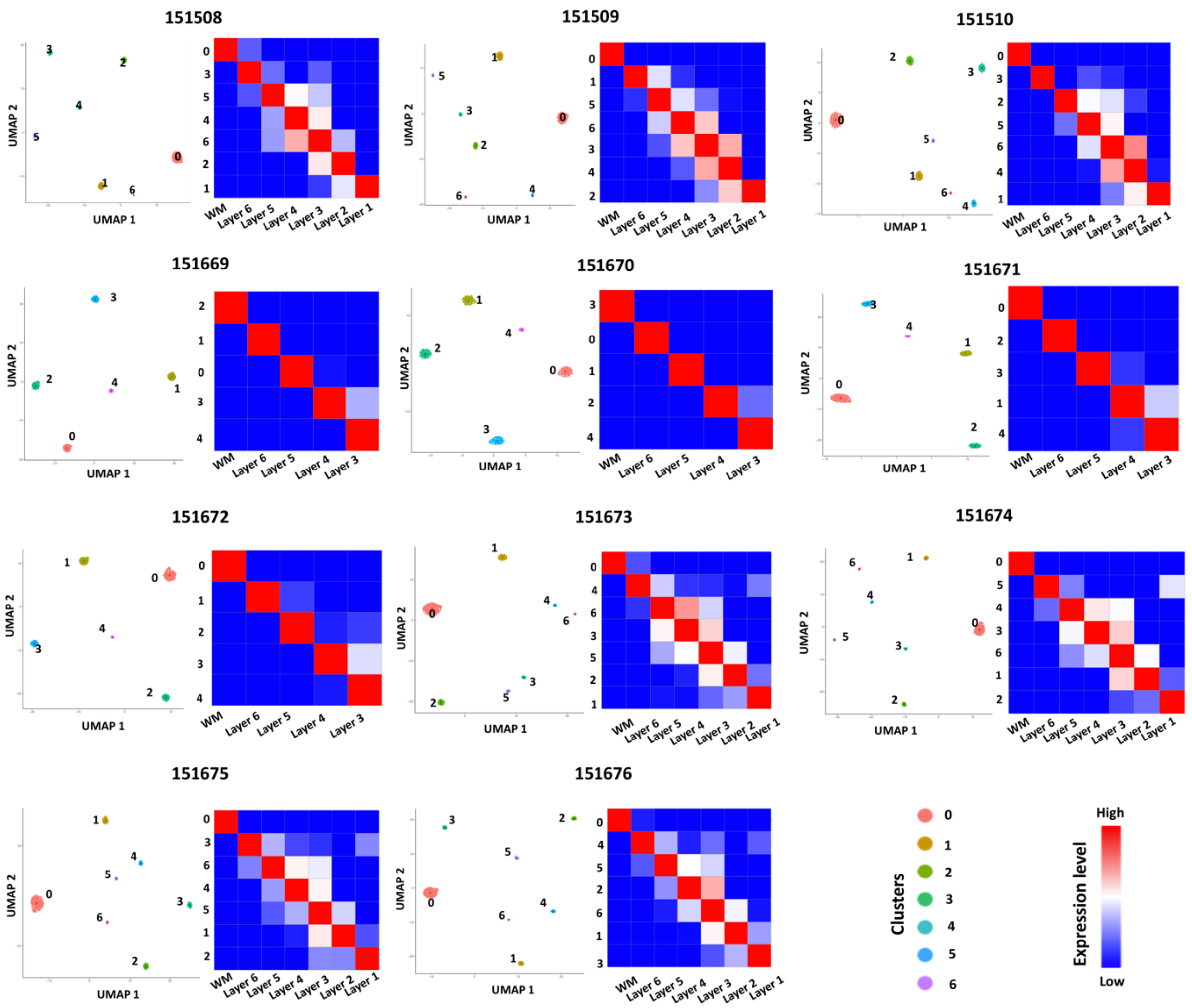

Supplementary Figure 2. stKeep is able to identify cluster-specific gene-modules on 11 slices of the human DLPFC dataset. For each slice, the left panel displays UMAP visualization of the identified gene-modules, and the right panel shows the mean gene expression of the identified gene-modules in different clusters. Source data are provided as a Source Data file.

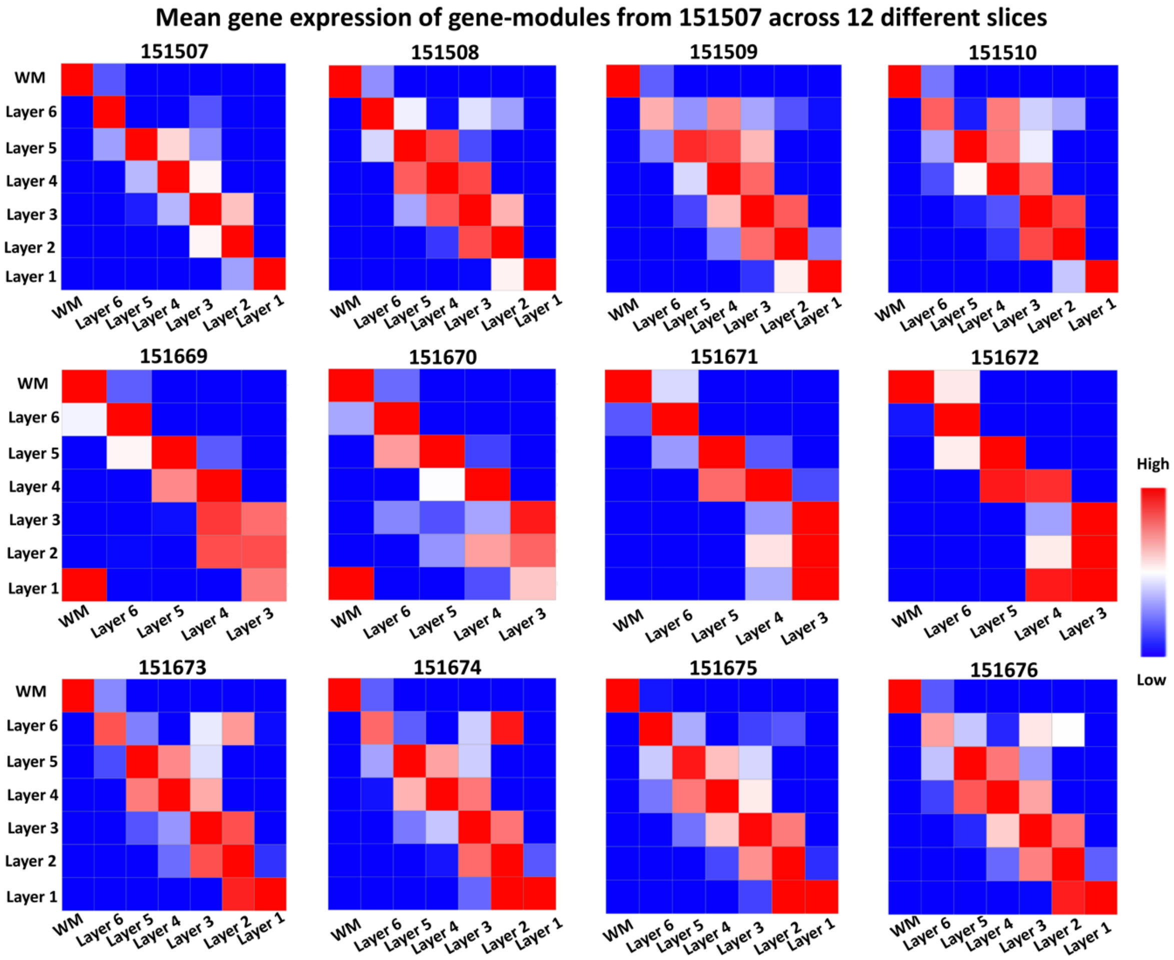

Supplementary Figure 3. The heatmap showing the mean gene expression of the seven gene-modules identified from slice 151507 on 12 different slices of the human DLPFC dataset. Note that gene expression data are normalized at both cell and gene levels. For each slice, rows and columns represent gene-modules and layers, respectively. Source data are provided as a Source Data file.

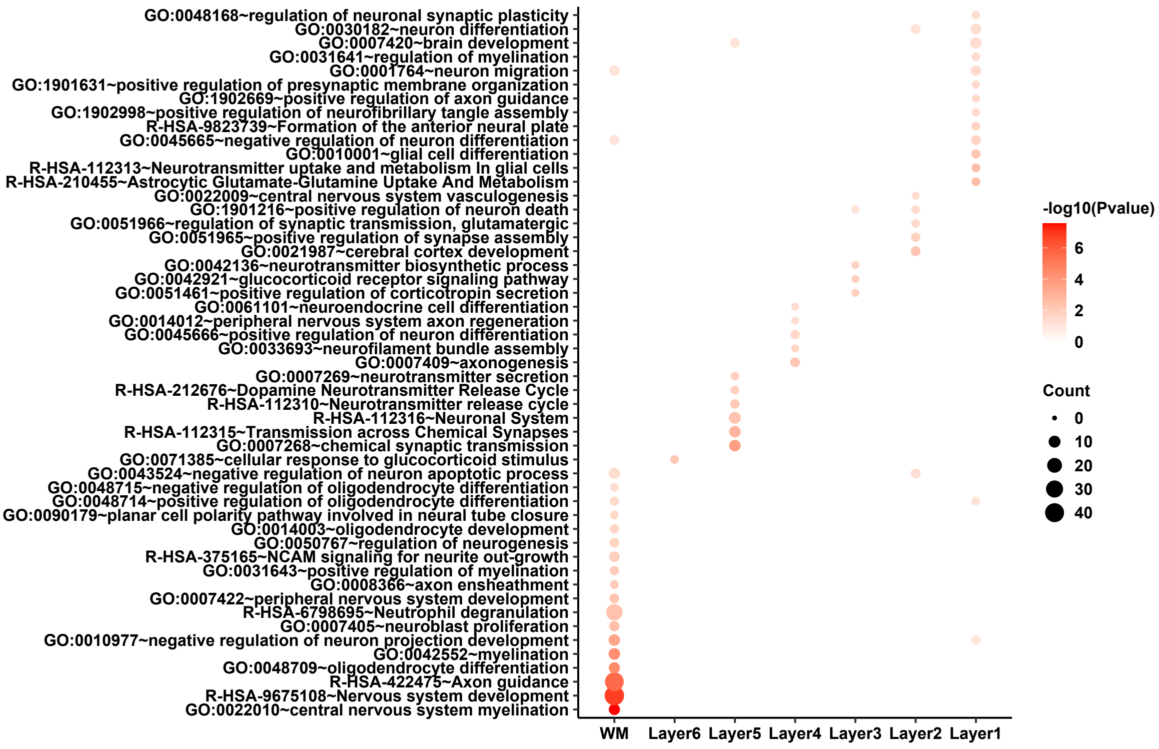

Supplementary Figure 4. Functional annotation of seven gene-modules identified from slice 151507 using DAVID online website (https://david.ncifcrf.gov/tools.jsp). For each annotation on each layer, the color and size indicate the $-log_{10}(p-value)$ and count, respectively. Unadjusted one-sided Fisher's exact test. Source data are provided as a Source Data file.

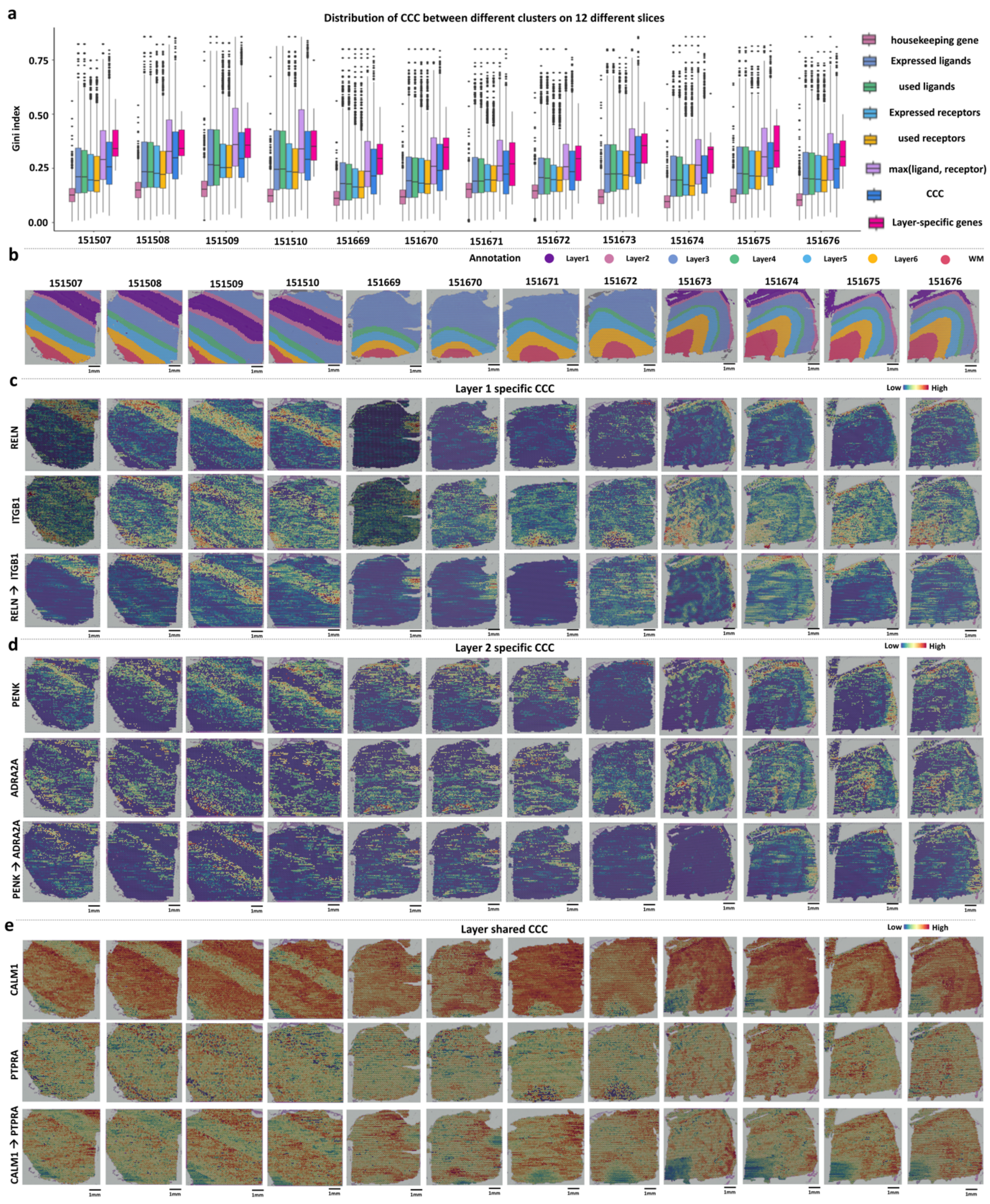

Supplementary Figure 5. stKeep can identify layer specific and shared CCC on 12 human DLPFC slices. a Boxplot displaying the GI score of interaction strengths of inferred CCC by stKeep on $n = 12$ slices, where we provide GI score of expression levels of the used ligands and receptors, maximum value of ligand and receptor for each LRP, all expressed ligands and receptors, housekeeping genes [4], and known layer-specific genes (see Supplementary Table 2) as a comparison. The number of LRPs, all expressed ligands and receptors, used ligands and

receptors, housekeeping genes, and known layer-specific genes is as follows: 155107 ($n =$ 2,585; 3,032; 3,554; 2,585; 2,585; 2,165; 15), 155108 ($n =$ 2,519; 2,973; 3,523; 2,519; 2,519; 2,164; 15), 155109 ($n =$ 2,608; 3,058; 3,556; 2,608; 2,608; 2,165; 15), 155110 ($n =$ 2,576; 3,006; 3,566; 2,576; 2,576; 2,164; 15), 151669 ($n =$ 2,528; 2,993; 3,490; 2,528; 2,528; 2,163; 15), 151670 ($n =$ 2,466; 2,939; 3,478; 2,466; 2,466; 2,163; 15), 151671($n =$ 2,654; 3,096; 3,574; 2,654; 2,654; 2,165; 15), 151672 ($n =$ 2,588; 3,068; 3,531; 2,588; 2,588; 2,164; 15), 151673 ($n =$ 2,662; 3,068; 3,102; 2,588; 2,588; 2,165; 15), 151674 ($n =$ 2,883; 3,248; 3,752; 2,883; 2,883; 2,165; 15), 151675 ($n =$ 2,618; 3,044; 3,573; 2,618; 2,618; 2,164; 15), 151676 ($n =$ 2,590; 3,034; 3,574; 2,590; 2,590; 2,164; 15). For each boxplot, the center line, box limits and whiskers separately indicate the median, upper and lower quartiles and 1.5 × interquartile range. **b** The manual annotation of six layers and WM on $n = 12$ slices of the human DLPFC dataset [3]. **c** Spatial expression of ligand *RELN* and receptor *ITGB1*, and their interaction strength on $n = 12$ slices. **d** Spatial expression of ligand *PENK* and receptor *ADRA2A*, and their interaction strength on 12 slices. **e** Spatial expression of ligand *CALM1* and receptor *PTPRA*, and their interaction strength on 12 slices. Source data are provided as a Source Data file.

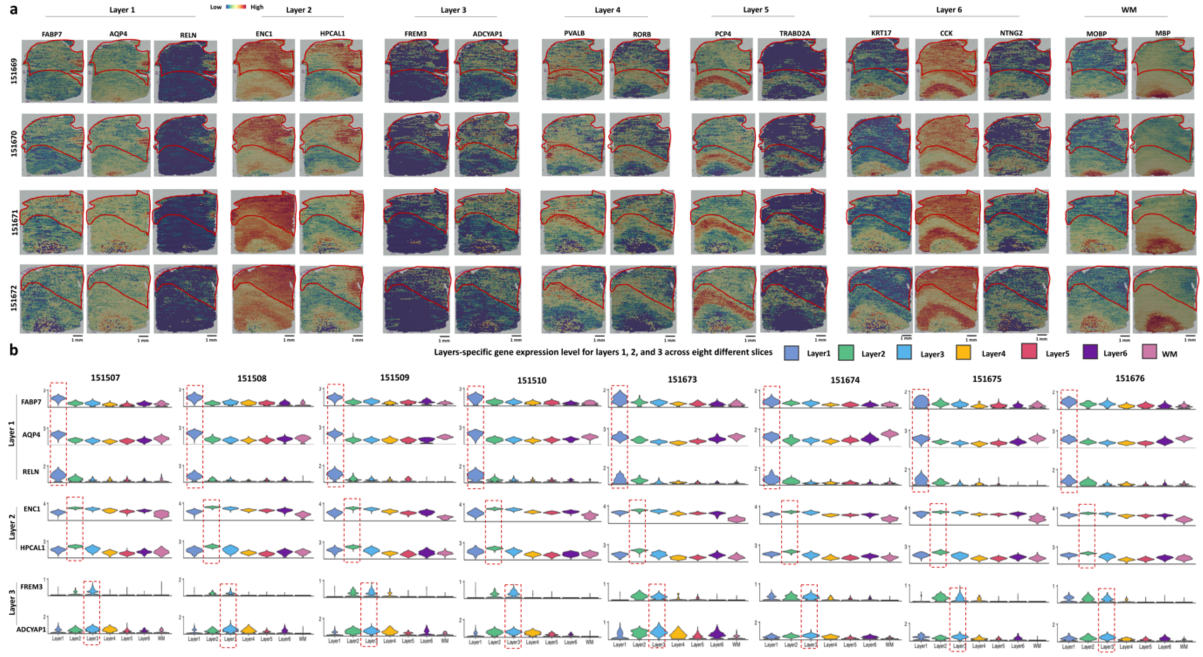

Supplementary Figure 6. Expression distribution of layer-specific genes on 12 slices of the human DLPFC dataset. a Spatial expression of known layer-specific genes on four slices from one experiment: 151669, 151670, 151671, and 151672. The red outline indicates the previously annotated Layer 3 [3]. It is important to note that *CCK* is annotated to Layer 2, Layer 3, and Layer 6, whereas being relatively over-expressed in Layer 6. Similarly, *ENC1* is annotated to Layer 2 and Layer 3 while exhibiting overexpression in Layer 2. **b** Violin plot illustrates gene expression of layer-specific genes for Layers 1, 2, and 3 across eight different slices: 151507-151510, and 151673-151676. Source data are provided as a Source Data file.

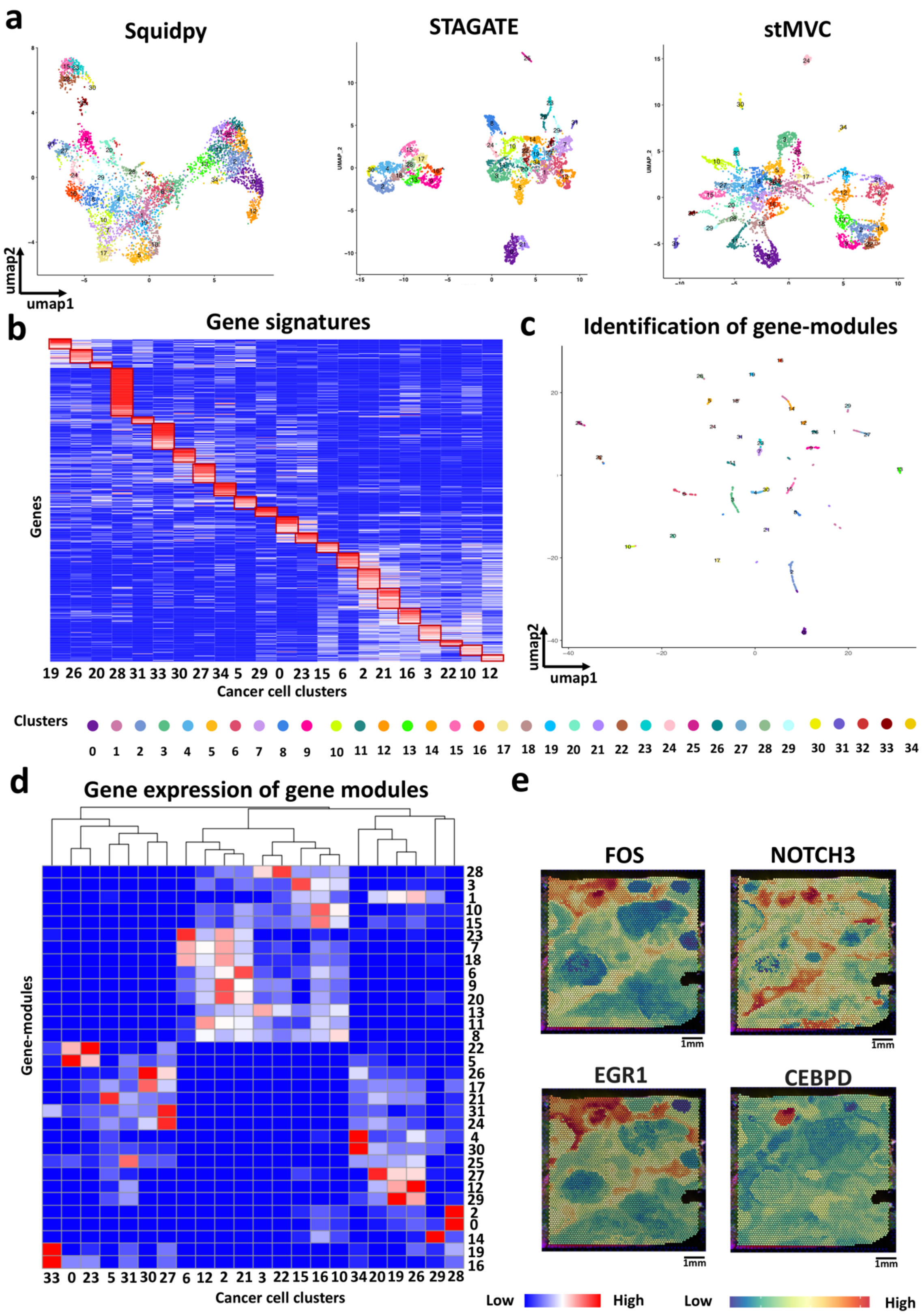

Supplementary Figure 7. Method comparison on the IDC sample (i.e., luminal B breast cancer). **a** UMAP visualization of the latent features by Squidpy, STAGATE, and stMVC, respectively. Each cluster is indicated by a color. **b** Heatmap of the mean gene expression of

signature genes across 22 spatial cancer clusters by stKeep. Rows and columns indicate signature genes and clusters. **c** UMAP visualization of the low-dimensional features for gene-modules by stKeep. Each color denotes a gene-module. **d** Heatmap of the mean gene expression of the identified gene-modules by stKeep. Rows and columns indicate gene-modules and clusters. **e** Spatial expression of key TFs (*FOS*, *NOTCH3*, *EGR1*, and *CEBPD*) in spatial cluster 28. Source data are provided as a Source Data file.

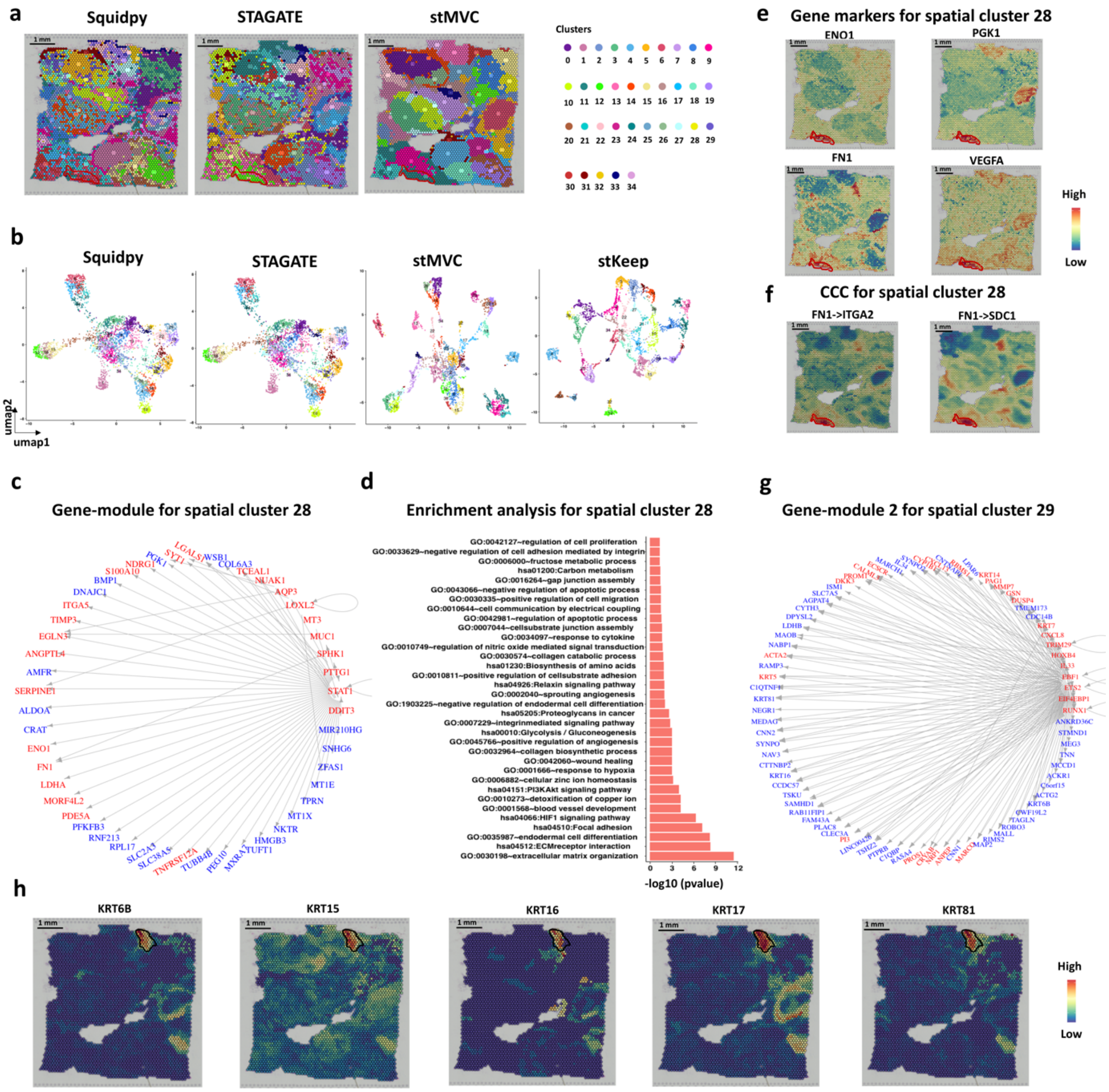

Supplementary Figure 8. Method comparison on the BAS1 (i.e., Her2^{+} breast cancer) sample. a Spatial clustering by Squidpy, STAGATE, and stMVC, respectively. **b** UMAP visualization of the latent features by Squidpy, STAGATE, stMVC, and stKeep, respectively. Each spatial cluster is indicated by a color. **c** The identified gene-module for spatial cluster 28 by stKeep. Regulator genes and target genes are highlighted in red and blue, respectively. **d** Functional annotation of two gene-modules for spatial cluster 28 by stKeep. Unadjusted one-sided Fisher's exact test. **e** Spatial expression of genes over-expressed in spatial cluster 28. **f** Spatial interaction strength between *FN1* and its two receptors. **g** The identified gene-module 2 for spatial cluster 29 by stKeep. **h** Spatial expression of basal genes: *KRT6B*, *KRT15*, *KRT16*, *KRT17*, and *KRT81*. Source data are provided as a Source Data file.

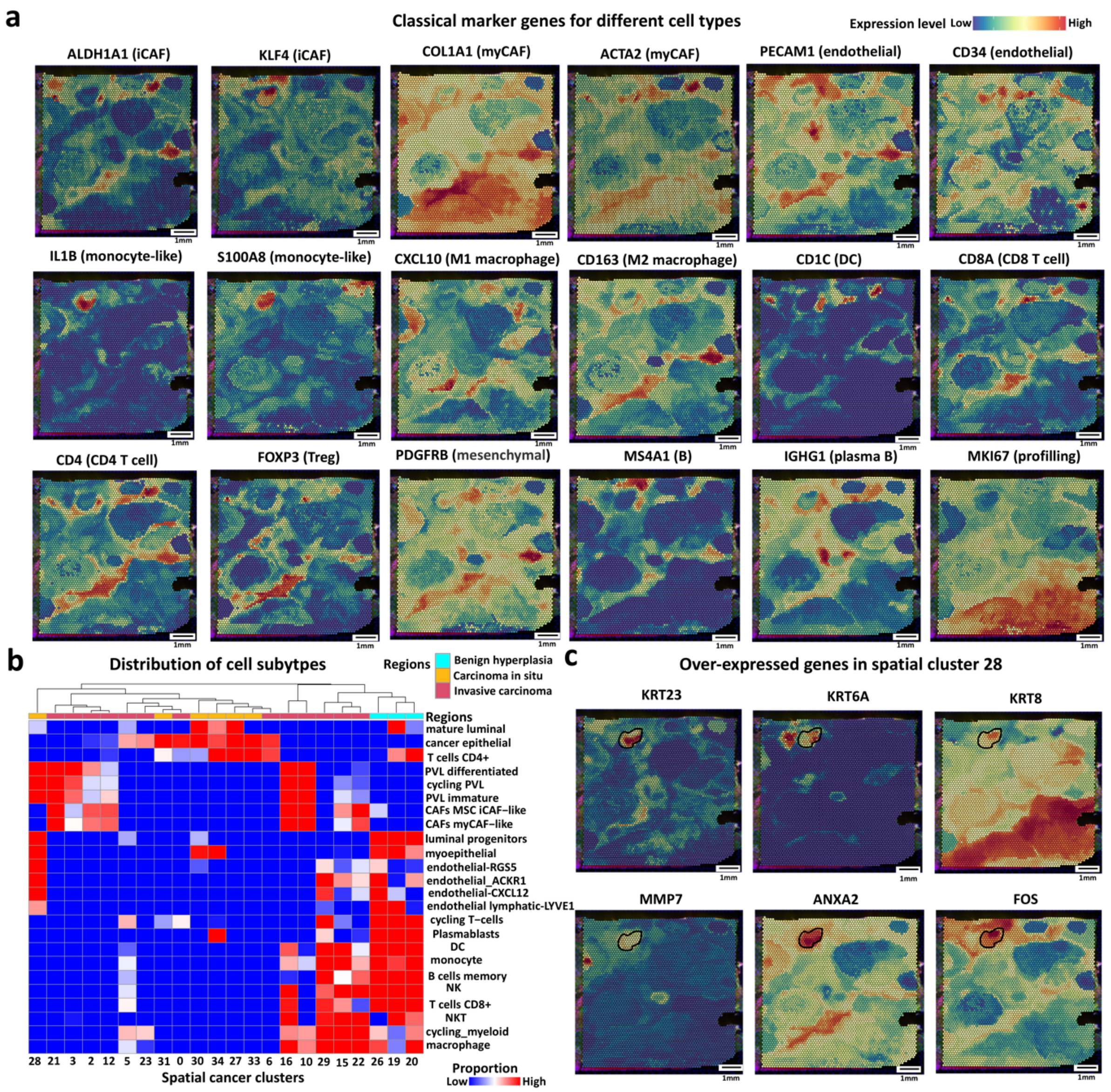

Supplementary Figure 9. The heterogeneity of TME on the IDC sample. a Spatial expression of classical marker genes for different cell types, including iCAF (*ALDH1A1*, and *KLF4*), myCAF (*COL1A1*, and *ACTA2*), endothelial (*PECAM1*, and *CD34*), monocyte-like (*IL1B*, and *S100A8*), M1 macrophage (*CXCL10*), M2 macrophage (*CD163*), DC (*CD1C*), CD8 T (*CD8A*), CD4 T (*CD4*), Treg (*FOXP3*), mesenchymal (*PDGFRB*), B (*MS4A1*), plasma B (*IGHG1*), profiling (*MKI67*). **b** Distribution of different cell subtypes in different spatial cancer clusters by GraphST [2]. **c** Spatial expression level of over-expressed genes (*KRT23*, *KRT6A*, *KRT8*, *MMP7*, *ANXA2*, and *FOS*) in spatial cluster 28. Source data are provided as a Source Data file.

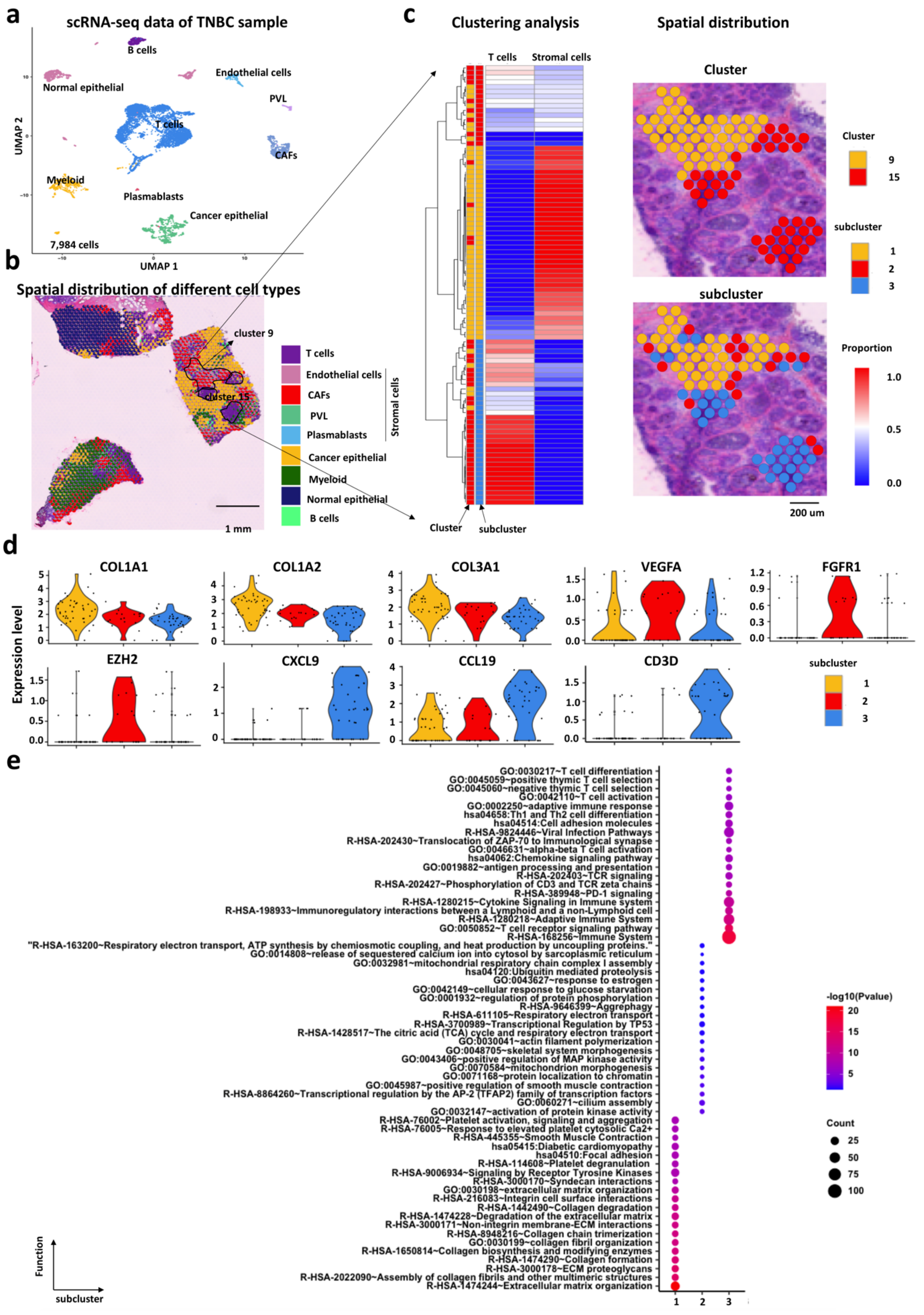

Supplementary Figure 10. Sub-clustering analysis on cluster 9 in the TNBC sample. a UMAP plot of nine different cell types of 7,984 cells from a TNBC sample. Each color denotes

a cell type. **b** Spatial proportions of nine cell types predicted by GraphST [2]. Each spot is a pie chart of the probability of its corresponding cell type. Each color indicates one cell type. **c** Clustering analysis of cell proportions of T and stromal cells within stroma, adipose and lymphocyte regions. **d** Violin plot showing marker gene expression across three subclusters: *COL1A1*, *COL1A2*, and *COL3A1* for subcluster 1; *VEGFA*, *FGFR1*, and *EZH2* for subcluster 2; and *CXCL9*, *CCL19*, and *CD3D* for subcluster 3. **e** Functional annotation of over-expressed genes in each subcluster using DAVID online website (https://david.ncifcrf.gov/tools.jsp). The color and size indicate $-log10(p-value)$ and count, respectively. Unadjusted one-sided Fisher's exact test. Source data are provided as a Source Data file.

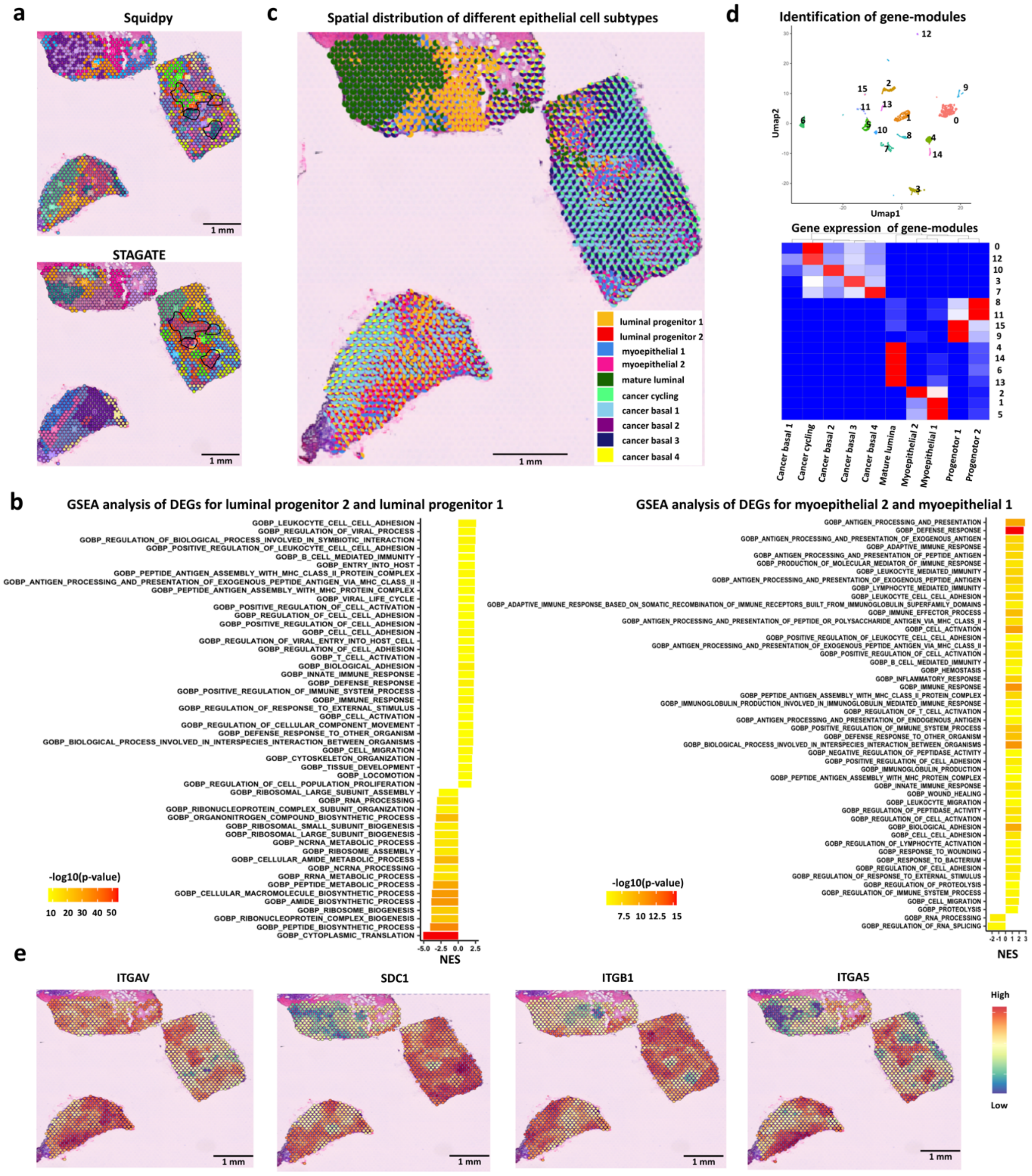

Supplementary Figure 11. Method comparison on the TNBC sample. a Spatial cluster predicted by Squidpy and STAGATE. **b** Gene set enrichment analysis (GSEA) of differentially expressed genes (DEGs) between luminal progenitor 2 and luminal progenitor 1 (left panel), as well as between myoepithelial 2 and myoepithelial 1 (right panel). The x-axis represents the normalized enrichment score (NES), and the color indicates $-log10(p-value)$. Unadjusted one-sided Kolmogorov-Smirnov test. **c** Spatial proportions of 10 distinct epithelial cell subtypes predicted by GraphST. Each spot is a pie plot of its corresponding cell type probabilities. Each color indicates one cell subtype. **d** UMAP visualization of the low-

dimensional features for gene-modules learned by stKeep (up panel). Each color denotes a gene-module. Heatmap of the mean gene expression of the identified gene-modules by stKeep (bottom panel). Rows and columns indicate gene-modules and 10 cell subtypes, respectively. **e** Spatial expression of four receptors for *COMP*. Source data are provided as a Source Data file.

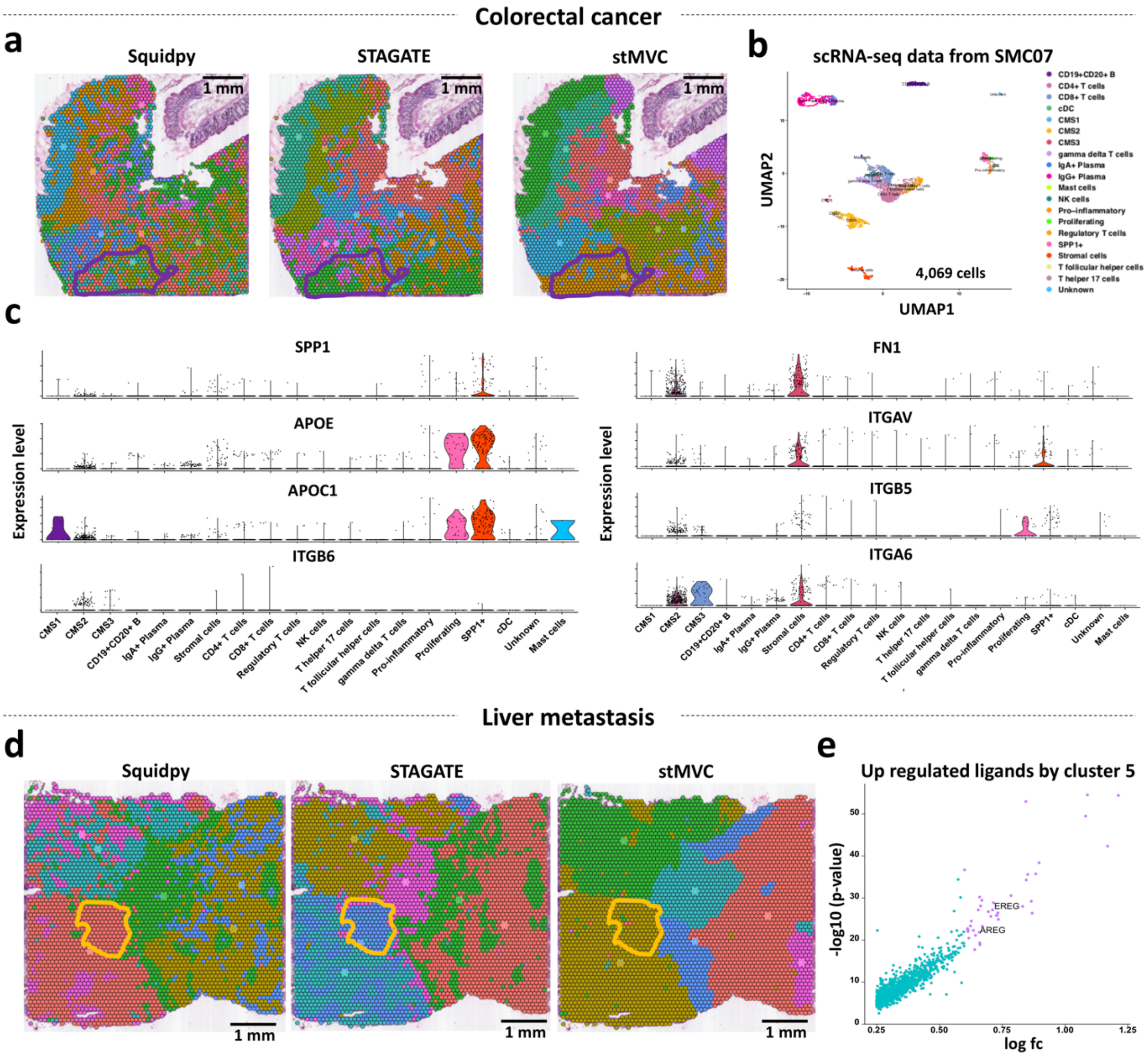

Supplementary Figure 12. Method comparison on the primary colorectal cancer (i.e., P1) and paired liver metastasis (i.e., LM1) samples. **a** Spatial cluster predicted by Squidpy, STAGATE, and stMVC, on P1 sample, respectively. **b** UMAP plot for 20 cell subtypes for 4,069 cells from one independent colorectal cancer sample [5]. Each color denotes one cell subtype. **c** Violin plot showing gene expression of representative genes including *SPP1*, *FN1*, *APOE*, *ITGAV*, *APOC1*, *ITGB1*, *ITGB6*, and *ITGA6* across different cell subtypes. **d** Spatial cluster predicted by Squidpy, STAGATE, and stMVC, on LM1 sample, respectively. **e** Scatter plot displaying the ligands over-expressed by spatial cluster 5. Unadjusted two-sided unpaired Wilcoxon test. Source data are provided as a Source Data file.

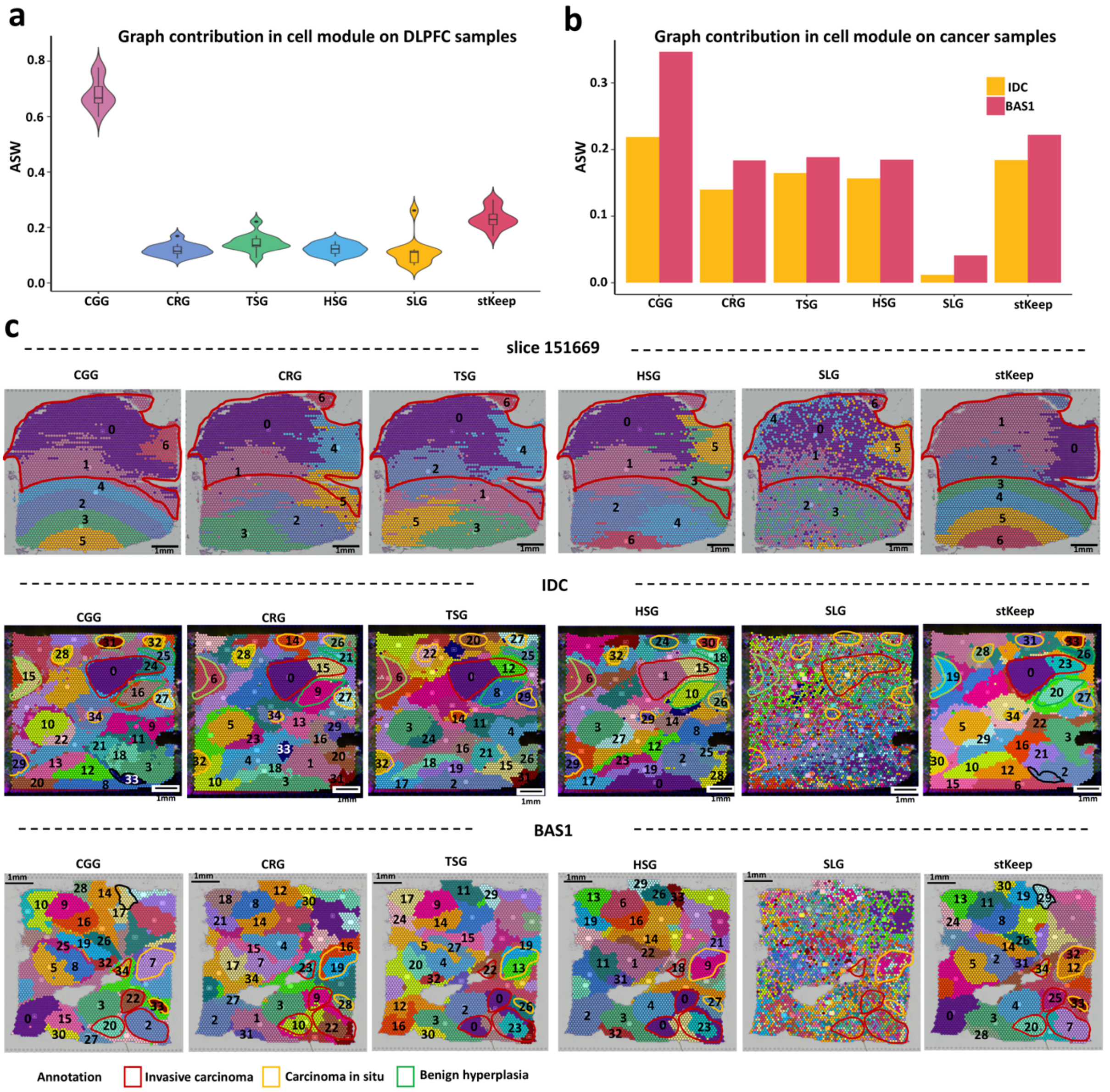

Supplementary Figure 13. Ablation analysis of graph component within the cell module. **a** Violin plot showing clustering score ASW used to evaluate the contribution of each graph within the cell-module on $n = 12$ human DLPFC slices. Specifically, after removing each graph, we predicted the spatial clustering based on the learned low-dimensional features. The lower the ASW, the higher the contribution of the graph. **b** Bar plot showing clustering score ASW used to evaluate the contribution of each graph within the cell-module on two cancer samples (IDC and BAS1). **c** Spatial clustering predicted by the low-dimensional features by removing each graph from stKeep on slice 151669, IDC, and BAS1 samples, where we also provide stKeep as a comparison. Note that spatial location graph, cell-region graph, histological similarity graph, transcriptomics similarity graph, and cell-gene graph are

abbreviated as SLG, CRG, HSG, TSG, and CGG, respectively. Source data are provided as a Source Data file.

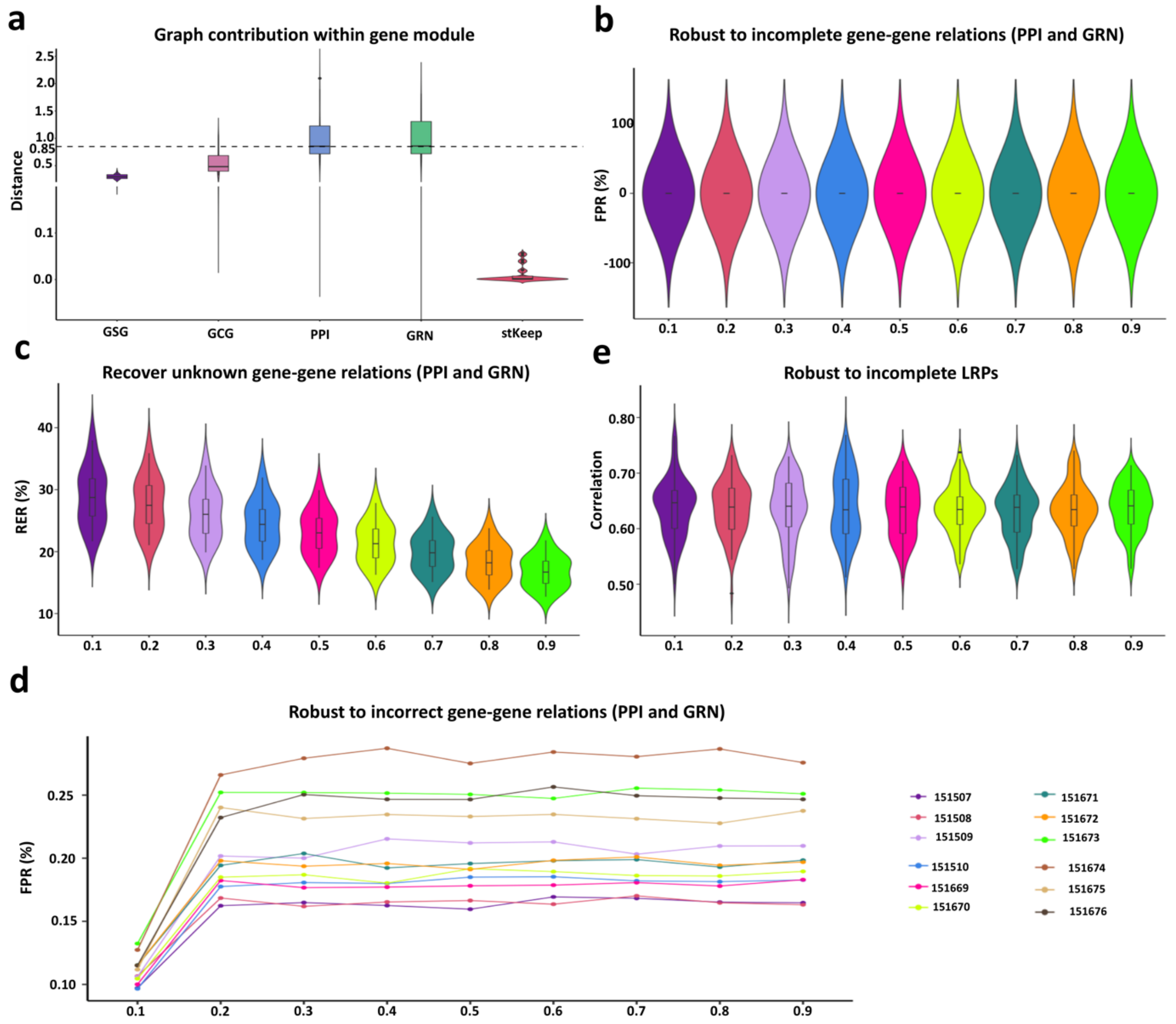

Supplementary Figure 14. Evaluation of the gene and CCC modules in stKeep on the 12 human DLPFC slices. a Violin plot showing distance of gene-pairs (identified by stKeep) to evaluate the contribution of each graph within the gene module. Specifically, after removing each graph, we calculated the distance of the gene-pairs based on the learned gene embedding. The shorter distance, the higher contribution of the graph. Note that gene-cell state graph and gene-cell graph are abbreviated as GSG and GCG, respectively. **b** Violin plot showing the false positive rate (FPR) of the learned gene embedding trained at different proportions of incomplete gene-gene interactions (PPI and GRN). **c** Violin plot showing the recover edge rate (RER) for the learned gene embedding trained at different scales of incomplete gene-gene interactions (PPI and GRN). **d** Line plot showing FPR of the learned gene embedding trained at different proportions of incorrect gene-gene interactions (PPI and GRN). Each color indicates a slice. **e** Violin plot showing Pearson correlation of the inferred CCC between spots

within a cluster, inferred for $n = 12$ slices with different proportion LRPs removed. Source data are provided as a Source Data file.

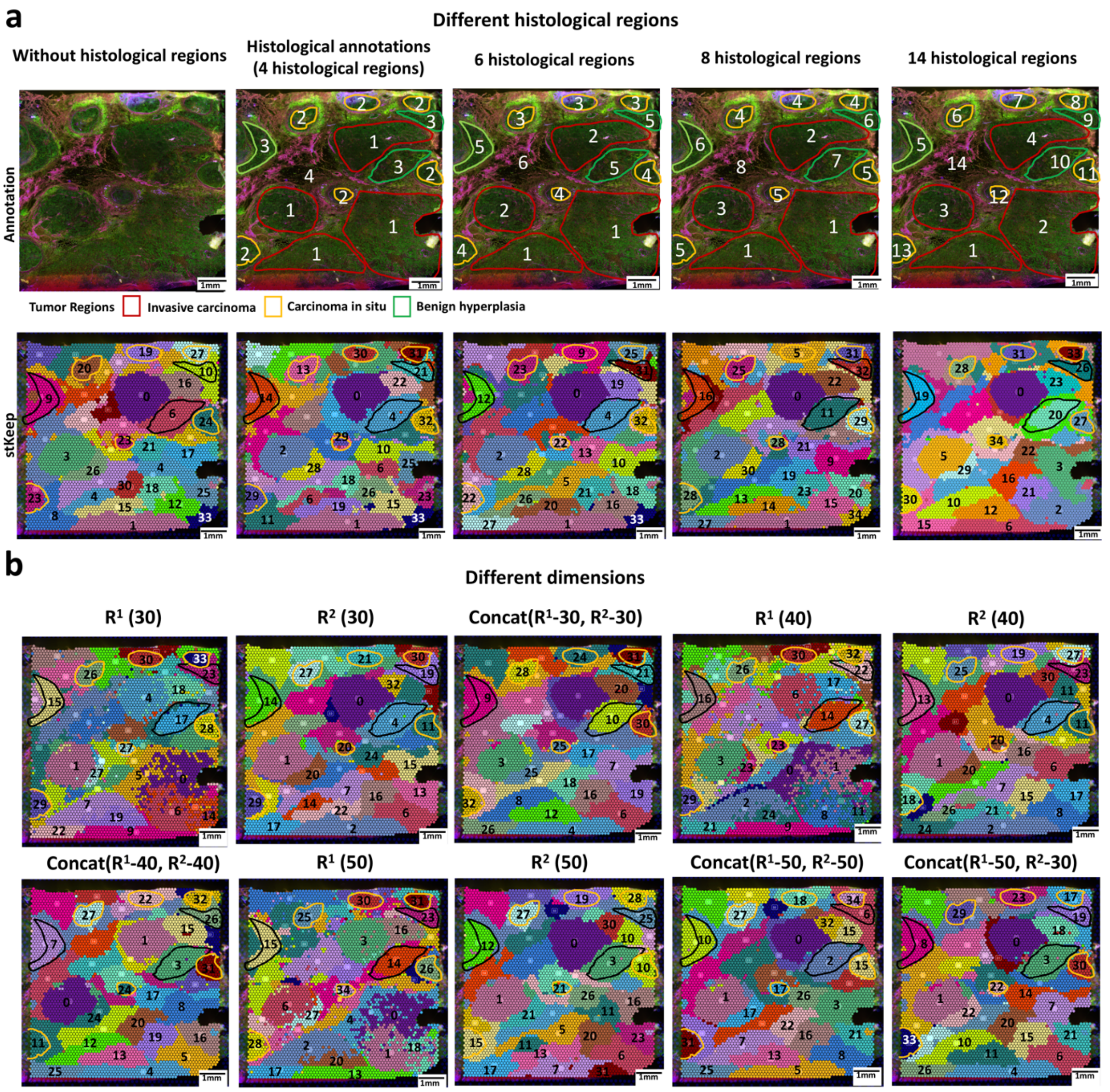

Supplementary Figure 15. Evaluation of spatial clustering with different numbers of histological regions and dimensional sizes on the IDC sample. a Spatial clustering by stKeep using varying numbers of histological regions. The top panel indicates the varied histological regions, while the bottom panel displays the corresponding spatial clustering results. In the model training without histological regions, positive samples are determined based on spatial nearest neighbors, while others are as negative samples. It is noteworthy that stKeep uses 14 histological regions as input for this analysis. **b** Spatial clustering predictions using the separately learned local hierarchical representations ($\mathbf{R}^1$) and global semantic representations ($\mathbf{R}^2$) with different dimensions (30, 40, and 50), as well as their simple concatenation (Concat ($\mathbf{R}^1, \mathbf{R}^2$)). Additionally, we present spatial clustering result by concatenating the optimal dimensions for $\mathbf{R}^1$ and $\mathbf{R}^2$. Source data are provided as a Source Data file.

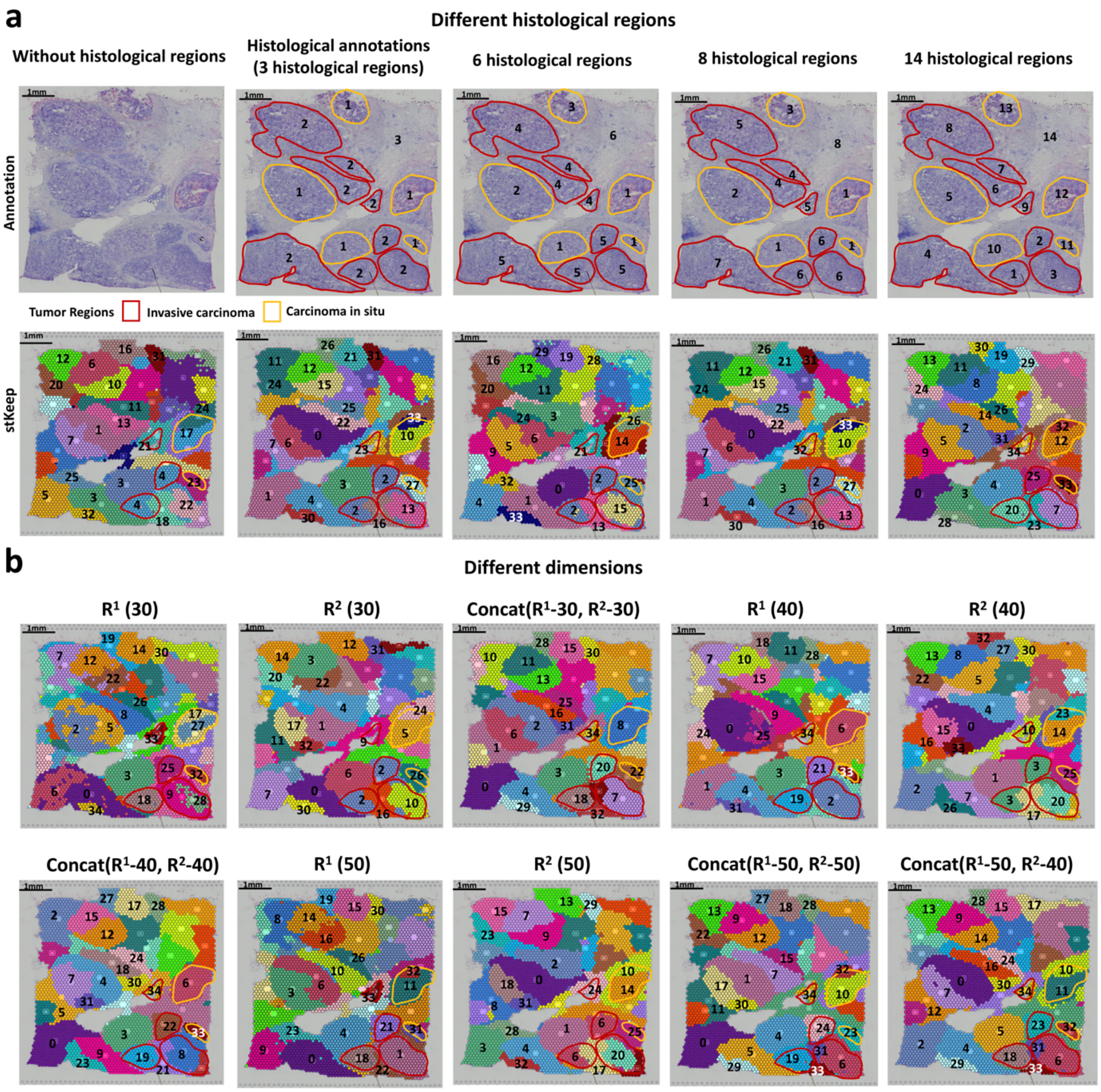

Supplementary Figure 16. Evaluation of spatial clustering with varying numbers of histological regions and dimensional sizes on the BAS1 sample. a Spatial clustering by stKeep using different numbers of histological regions. The top panel indicates the varied different numbers of histological regions, while the bottom panel displays the corresponding spatial clustering results. In the model training without histological regions, positive samples are determined based on spatial nearest neighbors, while others are as negative samples. It is noteworthy that stKeep uses 14 histological regions as input for this analysis. **b** Spatial clustering predictions using the separately learned local hierarchical representations ($\mathbf{R}^1$) and global semantic representations ($\mathbf{R}^2$) with different dimensions (30, 40, and 50), as well as their simple concatenation (Concat ($\mathbf{R}^1, \mathbf{R}^2$)). Additionally, we present spatial clustering result by

concatenating the optimal dimensions for $\mathbf{R}^1$ and $\mathbf{R}^2$. Source data are provided as a Source Data file.

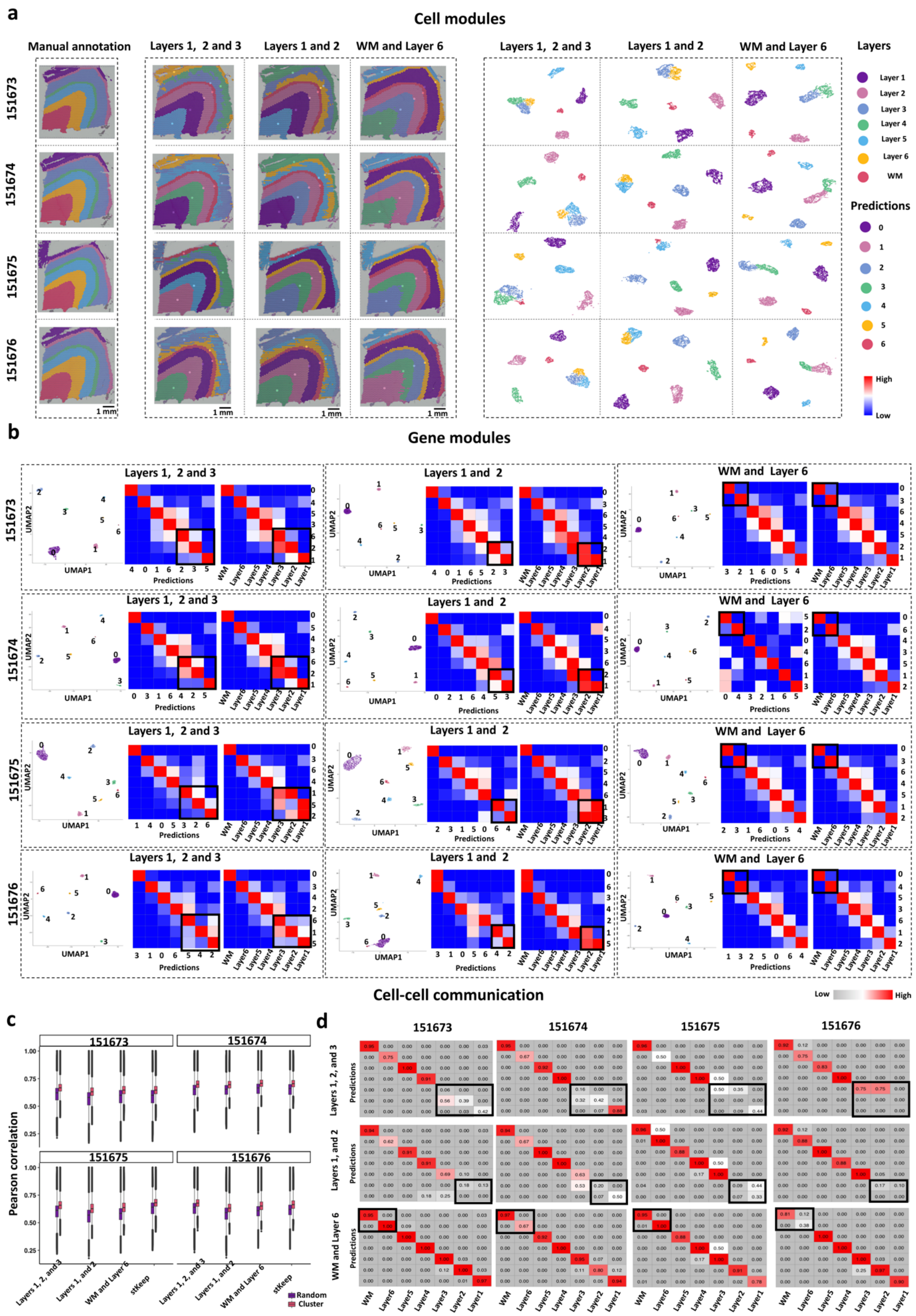
a
Cell modules
Manual annotation
Layers 1, 2 and 3
Layers 1 and 2
WM and Layer 6
151673
151674
151675
151676
1 mm
Layers
Layer 1
Layer 2
Layer 3
Layer 4
Layer 5
Layer 6
WM
Predictions
0
1
2
3
4
5
6
High
Low
b
Gene modules
UMAP1
UMAP2
Predictions
WM
Layer6
Layer5
Layer4
Layer3
Layer2
Layer1
Cell-cell communication
Low
High
c
Pearson correlation
1.00
0.75
0.50
0.25
Layers 1, 2, and 3
Layers 1, and 2
WM and Layer 6
stKeep
Random
Cluster
d
Layers 1, 2, and 3
Layers 1, and 2
WM and Layer 6

Supplementary Figure 17. The evaluation of histological regions on cell modules, gene modules, and CCC modules in the four human DLPFC slices. a Spatial clustering by stKeep using varying numbers of histological regions, i.e., five regions (WM, Layer 6, Layer 5, Layer 4, and a region containing Layers 3, 2, and 1), six regions (WM, Layer 6, Layer 5, Layer 4, Layer 3, and a region containing Layers 2 and 1), and six regions (Layer 5, Layer 4, Layer 3, Layer 2, Layer 1, and a region containing WM and Layer 6). The left and right panels indicate spatial distribution and UMAP visualization of the prediction. **b** Identification of gene-modules through the predicted spatial clustering results trained under different numbers of histological regions. For each slice, the left, middle, and right panels represent the UMAP visualization, mean gene expression of the identified gene-modules in different clusters, mean expression of the identified gene-modules in different annotated layers, respectively. **c** Boxplot showing Pearson correlation of interaction strength between $n = 1{,}000$ spot pairs within each cluster, inferred by stKeep under different histological regions for four slices, where we also provide the $n = 1{,}000$ randomly selected spot pairs for comparison. For each boxplot, the center line, box limits and whiskers separately indicate the median, upper and lower quartiles and $1.5 \times$ interquartile range. **d** Heatmap showing the ratio of the identified cluster-specific CCCs to layer-specific CCCs inferred by stKeep. Source data are provided as a Source Data file.

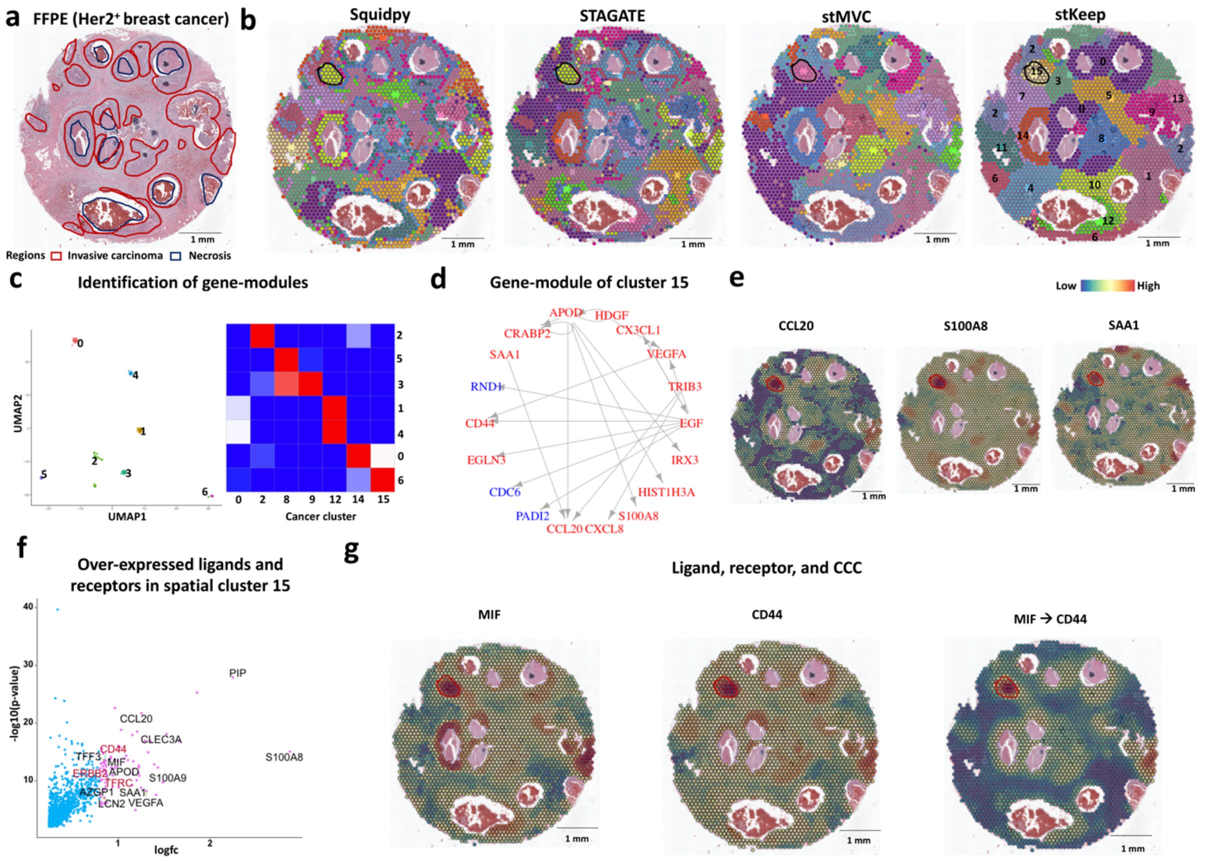

Supplementary Figure 18. Method comparison on the FFPE (Her2+ breast cancer) sample. a H&E tissue plot displaying manual annotation of 19 tumor regions from the 10X Genomics website (https://www.10xgenomics.com/resources/datasets/human-breast-cancer-ductal-carcinoma-in-situ-invasive-carcinoma-ffpe-1-standard-1-3-0). In data preprocessing, we adopted the previous recommendation [6] and removed 279 necrotic cells. **b** Spatial clustering predicted by Squidpy, STAGATE, stMVC, and stKeep. **c** The identified gene-modules in seven spatial cancer clusters. UMAP visualization presented on the left panel, while the right panel exhibits mean gene expression of gene-modules across different clusters. **d** The identified gene-module for spatial cluster 15 by stKeep, with regulator genes in red and target genes in blue. **e** Spatial expression of genes over-expressed in spatial cluster 15. **f** Scatter plot displaying the ligands (black) and receptors (red) over-expressed in spatial cluster 15. Unadjusted two-sided unpaired Wilcoxon test. **g** Spatial expression of ligand *MIF* and receptor *CD44*, along with their corresponding CCC interaction strengths. Source data are provided as a Source Data file.

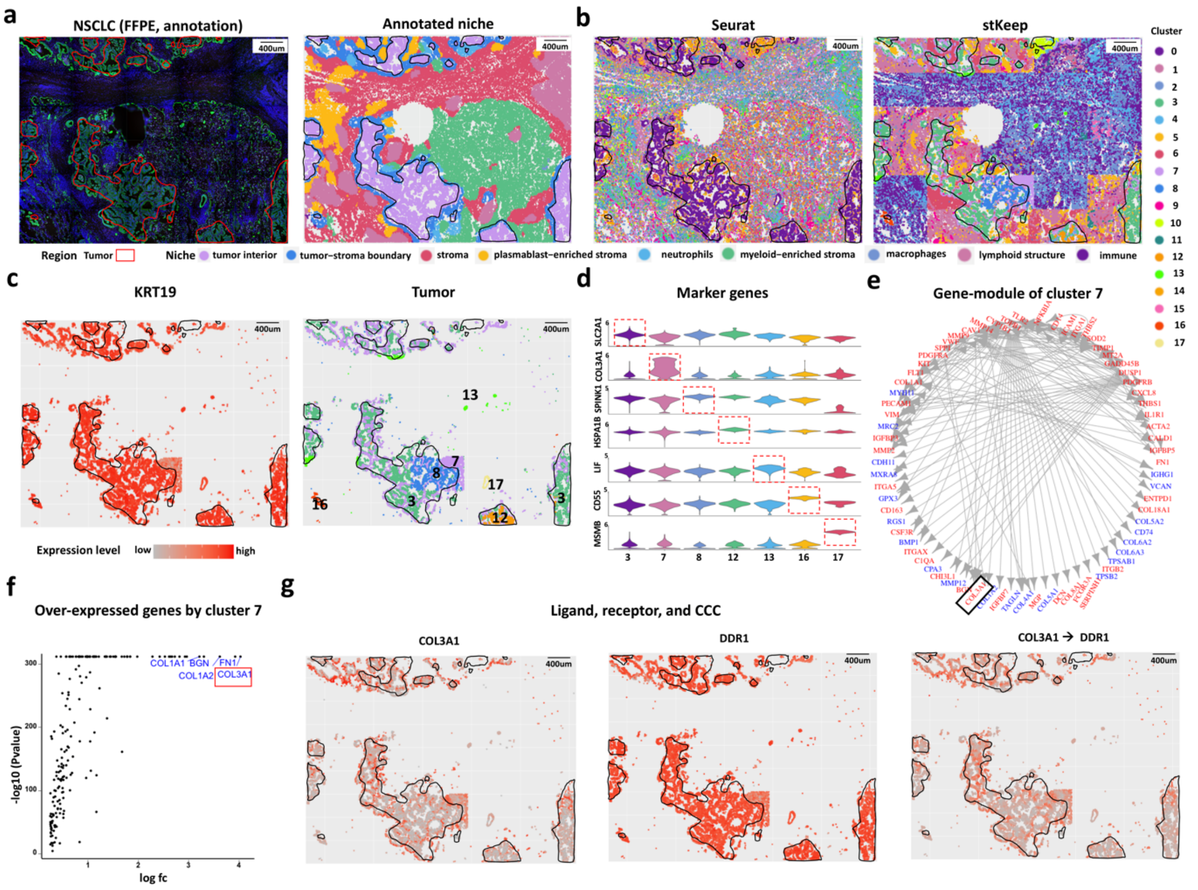

Supplementary Figure 19. Method comparison on the NSCLC (FFPE) sample by NanoString technology. a IF staining image showing manual annotation of 17 tumor regions (red outline), and niches from the NanoString website (https://nanostring.com/products/cosmx-spatial-molecular-imager/ffpe-dataset/nsclc-ffpe-dataset/). The intensity of DAPI (cell nuclei), PanCK (tumor cells), CD45 (leucocytes), and CD3 (T cells) is indicated by blue, green, yellow, and red, respectively. **b** Spatial clustering predicted by Seurat and stKeep. **c** Spatial expression of *KRT19* for epithelial cells (left panel). Spatial clusters within tumor regions (right panel). **d** Violin plot displaying expression levels of *SLC2A1*, *COL3A1*, *SPINK1*, *HSPA1B*, *LIF*, *CD55*, and *MSMB* across seven clusters. Each color indicates one cluster. **e** The identified gene-module for spatial cluster 7. The regulator and its target genes are colored in red and blue, respectively. Here, we displayed the over-expressed genes in spatial cluster 7 compared to other clusters, with a log_2fc greater than 3. **f** Scatter plot displaying the over-expressed genes in spatial cluster 7. Unadjusted two-sided unpaired Wilcoxon test. **g** Spatial expression of ligand *COL3A1* and receptor *DDR1*, along with their corresponding CCC interaction strengths. The black outline in **a**, **b**, **c**, and **g** indicates tumor regions, consistent with the red color in **a**. Source data are provided as a Source Data file.

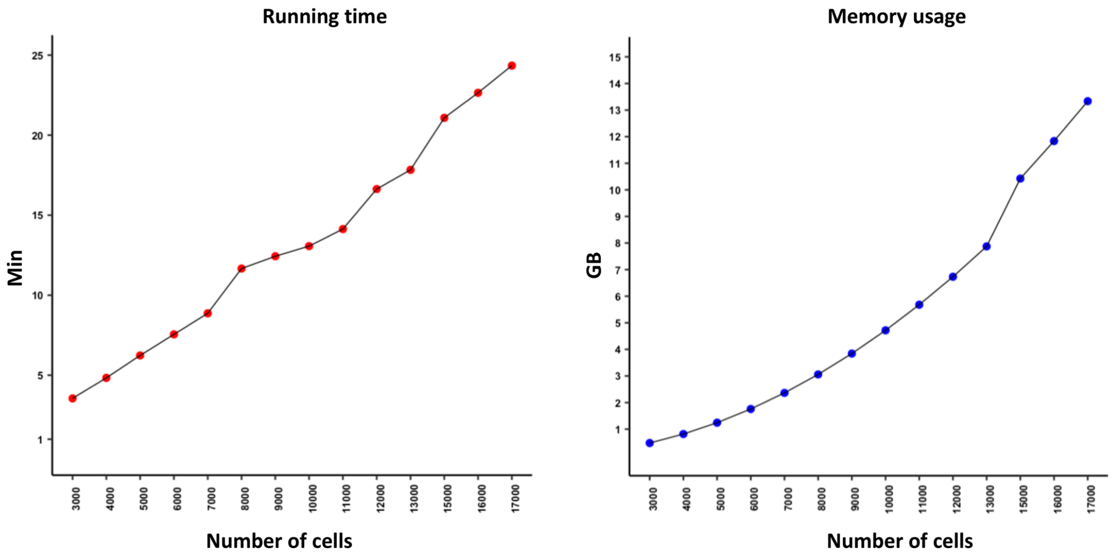

Supplementary Figure 20. Comparison of running time and memory usage for the training of stKeep model on the different numbers of cells by subsampling from the NSCLC sample. The experiments were tested on a GPU server with two NVIDIA Tesla V100 GPUs addressing 64GB. Source data are provided as a Source Data file.

<table>
<tr><th>SRT technology</th><th>Dataset name</th><th>Sample type</th><th>#Replicates</th><th>Slice</th><th>#Spots / cells</th><th>#Genes</th><th>Reference</th></tr>
<tr><td rowspan="18">Visium (fresh frozen)</td><td rowspan="12">DLPFC</td><td rowspan="12">Human brain dorsolateral prefrontal cortex</td><td rowspan="4">4</td><td>151507</td><td>4,226</td><td>>20,000</td><td rowspan="12">3</td></tr>
<tr><td>151508</td><td>4,384</td><td>>20,000</td></tr>
<tr><td>151509</td><td>4,789</td><td>>20,000</td></tr>
<tr><td>151510</td><td>4,634</td><td>>20,000</td></tr>
<tr><td rowspan="4">4</td><td>151669</td><td>3,661</td><td>>20,000</td></tr>
<tr><td>151670</td><td>3,498</td><td>>20,000</td></tr>
<tr><td>151671</td><td>4,110</td><td>>20,000</td></tr>
<tr><td>151672</td><td>4,015</td><td>>20,000</td></tr>
<tr><td rowspan="4">4</td><td>151673</td><td>3,639</td><td>>20,000</td></tr>
<tr><td>151674</td><td>3,673</td><td>>20,000</td></tr>
<tr><td>151675</td><td>3,592</td><td>>20,000</td></tr>
<tr><td>151676</td><td>3,460</td><td>>20,000</td></tr>
<tr><td>IDC</td><td>Luminal B breast cancer</td><td>1</td><td>IDC</td><td>4,727</td><td>>20,000</td><td>[7]</td></tr>
<tr><td>BAS1</td><td>Her2+ breast cancer</td><td>1</td><td>BAS1</td><td>3,798</td><td>>20,000</td><td>[8,9]</td></tr>
<tr><td>TNBC</td><td>Triple-negative breast cancer</td><td>1</td><td>CID44971</td><td>1,162</td><td>>20,000</td><td>[10]</td></tr>
<tr><td rowspan="2">P1 & LM1 (same patient)</td><td>Primary colorectal cancer</td><td>1</td><td>P1</td><td>2,917</td><td>>20,000</td><td rowspan="3">[11]</td></tr>
<tr><td>Matched liver metastasis</td><td>1</td><td>LM1</td><td>3,826</td><td>>20,000</td></tr>
<tr><td>LM2</td><td>Liver metastasis</td><td>1</td><td>LM2</td><td>3,721</td><td>>20,000</td></tr>
<tr><td>Visium (FFPE)</td><td>FFPE (Her2+ breast cancer)</td><td>Her2+ breast cancer</td><td>1</td><td>FFPE (Her2+ breast cancer)</td><td>2,239</td><td>>20,000</td><td>[12]</td></tr>
<tr><td>NanoString (FFPE)</td><td>NSCLC (FFPE)</td><td>Non-small cell lung cancer</td><td>1</td><td>NSCLC (FFPE)</td><td>98,002</td><td>980</td><td>[13]</td></tr>
</table>

Supplementary Table 1. Description of all used SRT datasets in this study.

**Layers**	**Genes**
Layer 1	*FABP7*, *AQP4*, *RELN*
Layer 2	*HPCAL1*
Layer 3	*CARTPT*, *FREM3*, *ADCYAP1*
Layer 4	*PVALB*, *RORB*
Layer 5	*TRABD2A*, *PCP4*
Layer 6	*KRT17*, *NTNG2*
WM	*MOBP*, *MBP*

Supplementary Table 2. Known layer-specific genes for the human DLPFC dataset from a previous study [3].

Technology	Sample	Cell modules						Gene modules			CCC modules
		Annotation	Squidpy	STAGTE	stMVC	Seurat	stKeep	#modules / clusters	#gene pairs	#significant gene pairs	#LRPs
Visium (fresh frozen)	151507	7	7	7	7	NA	7	7 / 7	12023	4934	2585
	151508	7	7	7	7	NA	7	7 / 7	14578	6474	2519
	151509	7	7	7	7	NA	7	7 / 7	11179	4152	2608
	151510	7	7	7	7	NA	7	7 / 7	9645	4287	2576
	151669	5	5	5	5	NA	5	5 / 5	11864	5911	2528
	151670	5	5	5	5	NA	5	5 / 5	12834	6046	2466
	151671	5	5	5	5	NA	5	5 / 5	15996	7256	2654
	151672	5	5	5	5	NA	5	5 / 5	15988	8108	2588
	151673	7	7	7	7	NA	7	7 / 7	14379	3331	2662
	151674	7	7	7	7	NA	7	7 / 7	14917	2150	2883
	151675	7	7	7	7	NA	7	7 / 7	13309	3463	2618
	151676	7	7	7	7	NA	7	7 / 7	12651	2953	2590
	IDC	14	35	35	35	NA	35	32 / 22	3454	2450	2681
	BAS1	14	35	35	35	NA	35	29 / 23	2694	1965	2994
	TNBC	16	22	22	22	NA	22	NA	NA	NA	2643
	P1	3	9	9	9	NA	9	NA	NA	NA	2702
	LM1	2	6	6	6	NA	6	NA	NA	NA	3190
	LM2	2	NA	NA	NA	NA	4	NA	NA	NA	2874
Visium (FFPE)	FFPE (Her2+ breast cancer)	19	16	16	16	NA	16	7 / 7	3630	3397	2910
NanoString (FFPE)	NSCLC (FFPE)	18	NA	NA	NA	18	18	8 / 7	5833	3391	828
scRNA-seq	CID44971	5	NA	NA	NA	10	NA	16 / 10	53149	50088	NA

Supplementary Table 3. Summary of all analyzed datasets in this study. NA indicates that there is no corresponding analysis result.

Tumor type	Sample	Cell types	Cell subtypes/states	#Cells	Marker genes	Reference
Breast cancer	Luminal B (scRNA-seq)	Epithelial cells	Cancer epithelial	6,223	*EPCAM*, *KRT19*	[10]
			Luminal progenitors	125	*ALDH1A3*	
			Myoepithelial	109	*KRT14*	
			Mature luminal	84	*FOXA1*	
		Lymphocytes	T cells CD8+	2,736	*CD8*	
			T cells CD4+	5,017	*CD4*	
			Cycling T−cells	177	*CD3D*, *MKI67*	
			NK	525	*AREG*	
			NKT	284	*FCGR3A*	
		Myeloid cells	Monocyte	268	*FCGR3A*, *S100A9*	
			Macrophage	698	*EGR1*, *CXCL10*	
			Cycling_myeloid	51	*MKI67*, *CD68*	
			DCs	226	*LAMP3*	
		B cells	B cells memory	267	*MS4A1*	
		Stromal cells	CAFs MSC iCAF−like	550	*KLF4*, *LEPR*	
			CAFs myCAF−like	869	*FAP*, *COL1A1*	
			PVL immature	485	*ALDH1A1*	
			PVL differentiated	1,028	*MYH11*	
			Cycling PVL	20	*ACTA2*, *MKI67*	
			Endothelial-ACKR1	703	*ACKR1*	
			Endothelial-RGS5	229	*RGS5*	
			Endothelial-CXCL12	425	*CXCL12*	
			Endothelial lymphatic-LYVE1	30	LYVE1	
		Plasma cells	Plasmablasts	451	*JCHAIN*	
		Total	24	21,580	32	
	TNBC (CID44971, scRNA-seq)	Epithelial cells	Mature luminal	169	*FOXA1*	
			Luminal progenitors	442	*ALDH1A3*	
			Myopeithelial cells	124	*KRT14*	
			Cancer basal cells	646	*VIM*	

			Cancer cycling cells	246	*MKI67*	
		Total	5	1,627	5	
	TNBC (CID44971, scRNA-seq)	Epithelial cells	Cancer epithelial	892	*EPCAM*, *KRT19*	
			Normal epithelial	735	*LTF*, *KRT8*	
		Stromal cells	Endothelial	217	*ENG*, *VWF*	
			CAFs	582	*DCN*, *COL1A2*	
			PVL	91	*ALDH1A1*	
		B cells	B cells	369	*MS4A1*	
		T cells	T cells	4,366	*CD3D*	
		Myeloid	Myeloid	684	*CD68*	
		Plasma cells	Plasmablasts	48	*JCHAIN*	
		Total	9	7,984	13	
Colorectal cancer	SMC07 (scRNA-seq)	Tumor cells	CMS1	8	*CASP7*, *CCND1*	5
			CMS2	493	*ASCL2*	
			CMS3	40	*TFF3, SLC26A3*	
		Stomal cells	Stomal cells	209	*APOE*	
		Plasma cells	IgA+ Plasma	106	*IGHA*	
			IgG+ Plasma	415	*IGHG*	
		B cells	CD19+CD20+ B	282	*CD19*, *MS4A1*, *IGHM*	
		Lymphocytes	CD4+ T cells	934	*CD4*, *IL7R*	
			CD8+ T cells	486	*CD8*	
			Regulatory T cells	264	*IL2RA*, *FOXP3*, *IL4R*	
			NK cells	89	*FCGR3A*, *KLRD1*	
			T helper 17 cells	146	*IL7A*, *IL17F*, *IL22*	
			Gamma delta T cells	160	*TRGC1*, *TRGC2*, *TRDC*	
			T follicular helper cells	80	*MAF*, *CXCL13*, *CXCR5*, *PDCD1*	
		Myeloid cells	SPP1+	140	*SPP1*	
			Pro-inflammatory	72	*IL1B*, *IL6*, *S100A8*, *S100A9*	
			Proliferation	32	*MKI67*, *STMN1*	

			cDC	32	*CD1C*, *FCER1A*	
			Unknown	79	*LILRA4*, *PTCRA*	
		Mast cells	Mast cells	2	*KIT*	
		Total	20	4,069	41	
Breast cancer	IDC(SRT)	Cancer tissue	Cluster 21	119	*SREBF1*, *PTK7*	NA
			Cluster 28	57	*KRT8*, *KRT18*, *CD44*	
	BAS1 (SRT)	Cancer tissue	Cluster 28	51	*STAT1*, *ENO1*, *PGK1*	
		Connective tissue (Cluster 29)	basal and myoepithelial cells	50	*KRT23, KRT6B*, *KRT5/14*, and *ACTA2*	
			CAFs		*COL1A1* and *COL1A2*	
			Endothelial cells		*ENG* and *VWF*	
			interleukins and chemokines		*CXCL8*, *CX3CL1*, *CCL17*, and *CXCL3*	
	TNBC (CID44971, scRNA-seq)	Epithelial cells	Luminal progenitor 1	373	*S100A1*, *PPP1R1B*, and *RGCC*	
			Luminal progenitor 2	65	*MMP7*, *KRT23*, and *SLC34A2*	
			myoepithelial 1	86	*C2orf40*, *MYH11*, *CNN1*, and *OXTR*	
			myoepithelial 2	37	*CDH13*, *COMP*, *THY1*, *FST*, *BGN*, *IGFBP6*, and *S100A2*	
			mature luminal	174	*TFF3*, *AGR3*, *TFF1*, *AFF3*, and *FBP1*	
			cancer cycling	135	*RRM2*, *CCNA2*, *UBE2C*, *CDK1*, *CDCA3*, *NUF2*, *MND1*, and *CDCA8*	
			cancer basal 1	316	*TMSB10*, *GDI2*, and *VIM*	
			cancer basal 2	162	*KIF1A*, *SLCO1A2*, and *DCLK1*	
			cancer basal 3	151	*SOX11*, *RNF144A*, *CTXN1*, *TTLL7*, and *GPC2*	
			cancer basal 4	128	*NINJ2*, *SLCO5A1*, and *GGACT*	

	FFPE (Her2+ breast cancer, SRT)	Cancer tissue	Cluster 15	19	*CL20*, *S100A8*, and *SAA1*	
Colorectal cancer and liver metastasis	P1 (SRT)	Cancer tissue	Cluster 6	260	*SPP1*, *FN1*, *APOE*, and *IFIT1*	
	LM1 (SRT)	Cancer tissue	Cluster 5	146	*AREG* and *EREG*	
Lung cancer	NSCLC (FFPE, SRT)	Cancer tissue	Cluster 3	9436	*SLC2A1*	
			Cluster 7	5671	*COL3A1*	
			Cluster 8	1692	*SPINK1*	
			Cluster 12	509	*HSPA1B*	
			Cluster 13	383	*LIF*	
			Cluster 16	138	*CD55*	
			Cluster 17	112	*MSMB*	

Supplementary Table 4. The detailed annotation of different cell types, subtypes or cell-states on the analyzed datasets. NA represents the marker gene discovered in our work.

References

1 Mark S. Handcock & Morris, M. *Relative Distribution Methods in the Social Sciences*. (Springer, 1999).

2 Long, Y. *et al.* Spatially informed clustering, integration, and deconvolution of spatial transcriptomics with GraphST. *Nat Commun* **14**, 1155 (2023).

3 Maynard, K. R. *et al.* Transcriptome-scale spatial gene expression in the human dorsolateral prefrontal cortex. *Nature neuroscience* **24**, 425-436 (2021).

4 Eisenberg, E. & Levanon, E. Y. Human housekeeping genes, revisited. *Trends Genet* **29**, 569-574 (2013).

5 Lee, H.-O. *et al.* Lineage-dependent gene expression programs influence the immune landscape of colorectal cancer. *Nature genetics* **52**, 594-603 (2020).

6 Rao, A., Barkley, D., França, G. S. & Yanai, I. Exploring tissue architecture using spatial transcriptomics. *Nature* **596**, 211-220 (2021).

7 Zhao, E. *et al.* Spatial transcriptomics at subspot resolution with BayesSpace. *Nature Biotechnology* **39**, 1375-1384 (2021).

8 Xun, Z. *et al.* Reconstruction of the tumor spatial microenvironment along the malignant-boundary-nonmalignant axis. *Nat Commun* **14**, 933 (2023).

9 Xu, C. *et al.* DeepST: identifying spatial domains in spatial transcriptomics by deep learning. *Nucleic Acids Res* **50**, e131-e131 (2022).

10 Wu, S. Z. *et al.* A single-cell and spatially resolved atlas of human breast cancers. *Nature genetics* **53**, 1334-1347 (2021).

11 Wu, Y. *et al.* Spatiotemporal immune landscape of colorectal cancer liver metastasis at single-cell level. *Cancer discovery* **12**, 134-153 (2022).

12 Genomics, X. *Human Breast Cancer: Ductal Carcinoma In Situ, Invasive Carcinoma (FFPE)*, <https://www.10xgenomics.com/resources/datasets/human-breast-cancer-ductal-carcinoma-in-situ-invasive-carcinoma-ffpe-1-standard-1-3-0> (2023).

13 Nanostring-Biostats. *CosMx SMI NSCLC FFPE Dataset*, <https://nanostring.com/products/cosmx-spatial-molecular-imager/ffpe-dataset/nsclc-ffpe-dataset/> (2023).
