# Supplementary material for: Dissecting tumor microenvironment from spatially resolved transcriptomics data by heterogeneous graph learning
Source: Nat Commun. 2024 Jun 13;15:5057. doi: 10.1038/s41467-024-49171-7 (PMC11176411; doi:10.1038/s41467-024-49171-7)
Supplement: Supplementary file 3 — Reporting Summary [file 41467_2024_49171_MOESM3_ESM.pdf]

nature portfolio

Corresponding author(s): Luonan Chen and Chunman Zuo

Last updated by author(s): Mar 24, 2024

Reporting Summary

Nature Portfolio wishes to improve the reproducibility of the work that we publish. This form provides structure for consistency and transparency in reporting. For further information on Nature Portfolio policies, see our Editorial Policies and the Editorial Policy Checklist.

Statistics

For all statistical analyses, confirm that the following items are present in the figure legend, table legend, main text, or Methods section.

n/a	Confirmed	
☐	☒	The exact sample size (*n*) for each experimental group/condition, given as a discrete number and unit of measurement
☐	☒	A statement on whether measurements were taken from distinct samples or whether the same sample was measured repeatedly
☐	☒	The statistical test(s) used AND whether they are one- or two-sided<br>*Only common tests should be described solely by name; describe more complex techniques in the Methods section.*
☒	☐	A description of all covariates tested
☐	☒	A description of any assumptions or corrections, such as tests of normality and adjustment for multiple comparisons
☐	☒	A full description of the statistical parameters including central tendency (e.g. means) or other basic estimates (e.g. regression coefficient) AND variation (e.g. standard deviation) or associated estimates of uncertainty (e.g. confidence intervals)
☐	☒	For null hypothesis testing, the test statistic (e.g. *F*, *t*, *r*) with confidence intervals, effect sizes, degrees of freedom and *P* value noted<br>*Give P values as exact values whenever suitable.*
☒	☐	For Bayesian analysis, information on the choice of priors and Markov chain Monte Carlo settings
☒	☐	For hierarchical and complex designs, identification of the appropriate level for tests and full reporting of outcomes
☐	☒	Estimates of effect sizes (e.g. Cohen's *d*, Pearson's *r*), indicating how they were calculated

Our web collection on statistics for biologists contains articles on many of the points above.

Software and code

Policy information about availability of computer code

Data collection	All data analyzed within the manuscript are publicly available. No additional software was utilized for the data collection process.
Data analysis	stKeep is open source and publicly available at https://github.com/cmzuo11/stKeep. Other tools and package used in the manuscript include: python 3.8.18, numpy 1.22.4, pandas 2.0.3, scipy 1.8.1, scikit-learn 1.3.2, torch 1.13.0, tqdm 4.51.0, scanpy 1.9.6, Pillow 9.5.0, seaborn 0.11.1, matplotlib 3.7.3, glob2 0.7, anndata 0.9.2, argparse 1.1, json 2.0.9, cv2 (opencv-python) 4.8.1.78, torchvision 0.14.0, STAGATE 1.0.1, Squidpy 1.2.2, stMVC 0.0.2, Seurat v4, ggplot2 3.3.6, igraph 1.3.2, and ggrepel 0.9.1.

For manuscripts utilizing custom algorithms or software that are central to the research but not yet described in published literature, software must be made available to editors and reviewers. We strongly encourage code deposition in a community repository (e.g. GitHub). See the Nature Portfolio guidelines for submitting code & software for further information.

Data

Policy information about availability of data

All manuscripts must include a data availability statement. This statement should provide the following information, where applicable:

- Accession codes, unique identifiers, or web links for publicly available datasets
- A description of any restrictions on data availability
- For clinical datasets or third party data, please ensure that the statement adheres to our policy

The raw count matrix, histology, and spatial location data for IDC, BAS1, and FFPE (Her2+ breast cancer) samples are publicly available at the 10X Genomics Website

(https://support.10xgenomics.com/spatial-gene-expression/datasets), while TNBC sample are available from the Zenodo data repository (https://doi.org/10.5281/zenodo.4739739). The human DLPFC dataset are available from the R package spatialLIBD (http://spatial.libd.org/spatialLIBD/). Primary colorectal cancer and two liver metastasis samples are available from the website: (http://www.cancerdiversity.asia/scCRLM/). The NSCLC sample are downloaded from website (https://nanostring.com/products/cosmx-spatial-molecular-imager/ffpe-dataset/nsclc-ffpe-dataset/). The scRNA-seq datasets of breast and colorectal cancers are publicly available from Gene Expression Omnibus database with GSE176078 [https://www.ncbi.nlm.nih.gov/geo/query/acc.cgi?acc=GSE176078] and GSE132465 [https://www.ncbi.nlm.nih.gov/geo/query/acc.cgi?acc=GSE132465], respectively. The C5: ontology gene sets are available from MSigDB database (https://www.gsea-msigdb.org/gsea/msigdb/human/genesets.jsp?collection=C5). The ligand-receptor interaction databases are located at CellChatDB (http://www.cellchat.org/cellchatdb/), ConnectomeDB (https://db.humanconnectome.org/), CellphoneDB (https://www.cellphonedb.org/), and NicheNet (https://zenodo.org/records/7074291). Source data provided with this paper, and are available at figshare.

Research involving human participants, their data, or biological material

Policy information about studies with human participants or human data. See also policy information about sex, gender (identity/presentation), and sexual orientation and race, ethnicity and racism.

Reporting on sex and gender	Not applicable.
Reporting on race, ethnicity, or other socially relevant groupings	Not applicable.
Population characteristics	Not applicable.
Recruitment	Not applicable.
Ethics oversight	Not applicable.

Note that full information on the approval of the study protocol must also be provided in the manuscript.

Field-specific reporting

Please select the one below that is the best fit for your research. If you are not sure, read the appropriate sections before making your selection.

☒ Life sciences ☐ Behavioural & social sciences ☐ Ecological, evolutionary & environmental sciences

For a reference copy of the document with all sections, see nature.com/documents/nr-reporting-summary-flat.pdf

Life sciences study design

All studies must disclose on these points even when the disclosure is negative.

Sample size	No sample size was selected. All data used in the manuscript were downloaded from public databases, serving to assess the ability of stKeep. We used (i) a human DLPFC dataset from the Visium platform (a spot-based technology), rich in known information such as cell annotation and layer-specific genes. This dataset validated the functionality of stKeep in dissecting tissue structure, identifying corresponding gene-modules and cell-cell communication (CCC) models; (ii) the human breast (Illumina B, Her2+, and triple-negative breast cancer), colorectal cancer and paired liver metastasis from Visium platform to validate that stKeep can identify local tumor domains and associated gene programs and CCC activities; (iii) FFPE (Her2+ breast cancer) sample from Visium (FFPE) technology to demonstrate the efficiency of stKeep in processing non-fresh SRT data; and (iv) non-small-cell lung cancer sample with ~100K cells from NanoString platform (single-cell resolution technology) to show the scalability of stKeep in processing these large-scale datasets. Collectively, these datasets sufficiently demonstrate stKeep's versatility, efficiency, and functional capabilities.
Data exclusions	All spots/cells and genes of each dataset was used, and no exclusion was done prior to analysis. We performed quality control and gene selection for spatially resolved transcriptomics data based on established standards in the filed.
Replication	We did not conduct biological or technical replications. All data were obtained from the public domain. The reproducibility details are outlined in the methods or GitHub section. To assess the stability and reproducibility of stKeep, we ran the algorithm five times on the dataset using the same parameters, and calculated the mean of the results.
Randomization	The allocation was random.
Blinding	Blinding is irrelevant as there was no data collection involved in the present study. Data information was included in the original publication.

Reporting for specific materials, systems and methods

We require information from authors about some types of materials, experimental systems and methods used in many studies. Here, indicate whether each material, system or method listed is relevant to your study. If you are not sure if a list item applies to your research, read the appropriate section before selecting a response.

Materials & experimental systems

n/a	Involved in the study
☒	☐ Antibodies
☒	☐ Eukaryotic cell lines
☒	☐ Palaeontology and archaeology
☒	☐ Animals and other organisms
☒	☐ Clinical data
☒	☐ Dual use research of concern
☒	☐ Plants

Methods

n/a	Involved in the study
☒	☐ ChIP-seq
☒	☐ Flow cytometry
☒	☐ MRI-based neuroimaging

Plants

Seed stocks	Not applicable.
Novel plant genotypes	Not applicable.
Authentication	Not applicable.
